# The Influence of Non-Optimal Rearing Conditions and Substrates on the Performance of the Black Soldier Fly (*Hermetia illucens*)

**DOI:** 10.3390/insects13070639

**Published:** 2022-07-16

**Authors:** Nuno Ribeiro, Rui Costa, Olga M. C. C. Ameixa

**Affiliations:** 1 Ecomare, Centre for Environmental and Marine Studies (CESAM), Department of Biology, University of Aveiro, 3810-193 Aveiro, Portugal; 2 Research Centre for Natural Resources, Environment and Society (CERNAS), Coimbra Agriculture School, Bencanta, 3045-601 Coimbra, Portugal; ruicosta@esac.pt; 3 Polytechnic Institute of Coimbra (IPC), Coimbra Agriculture School, Bencanta, 3045-601 Coimbra, Portugal

**Keywords:** black soldier fly, alternative proteins, bioconversion, insects feed, circular economy

## Abstract

**Simple Summary:**

The black soldier fly is one of the insect species most frequently reared as an alternative protein source. Even though many advances have been made in the last decade regarding environmental and process conditions, there are still several gaps that can delay the upscaling of industrial production systems. One of such gaps is related to the effect of suboptimal feeding regimes of mono-waste streams. This research aims to assess the development and bioconversion behaviour of black soldier fly larvae under suboptimal conditions. It was observed that specific types of vegetable and fruit wastes, such as apple, spinach and grape pomace, may contribute to achieve low insect biomass yields and, thus, reduce the efficiency of industrial operations.

**Abstract:**

Among the insect species reared as alternative protein sources, *Hermetia illucens* (black soldier Fly, BSF) has shown a huge potential mostly due to its high protein content, its bioconversion rates, and versatility in using different feeding substrates. Insect rearing may use continuous or batch feeding regimes and, among the used substrates, supermarket feedstock waste has gained recent interest under a circular economy perspective, but several uncertainties remain regarding the heterogeneity and the potential effects of the quantity and quality of these substrates on BSF larvae (BSFL) development. In this experimental work, five replicates of a hundred BSFL were fed in a continuous feeding regime, using seven different isolated vegetables as substrates (wheat bran, pumpkin, apple, grape pomace, red onion, red cabbage, and spinach), at three different temperatures (20, 25, and 30 °C) and two substrate moisture conditions (natural and 70% substrate moisture), until 50% of the larvae achieved the prepupal stage. BSFL performance and bioconversion parameters were evaluated. Our results show that some substrates should be avoided when rearing *Hermetia illucens* on feedstocks. Among these, apple feed led to poorer and slower development performances with more than 100 days of larval stage, while grape pomace and spinach showed higher mortality rates, which may be due to some anti-nutritional compounds. Larvae fed on pumpkin, red cabbage, and red onion presented good bioconversion results with higher values of efficiency of conversion of digested feed between 14.4 and 25. This work delivers relevant results for black soldier fly reared on a continuous feeding system using vegetable feedstock substrates and their potential trade-offs.

## 1. Introduction

Global demographical trends continue to exert an increasing demand for food, accompanied by unsustainable pressure on natural resources, which lead to the development of new protein sources and alternative food and feed production systems [1]. Among the several alternative protein sources that have been developed in recent years, plant-based ingredients, microalgae, or insect-based proteins [2] have been gaining stakeholders’ attention. In fact, an entire new market for the production and commercialization of insect protein has been developed as well as the accompanying necessary technology and implementation of industrial-scale rearing facilities worldwide [3].

One of the most successful insect species reared at an industrial scale is *Hermetia illucens* L. due to its versatility in feeding on different substrates. Over the last decade, several studies have been conducted regarding the characteristics of BSFL [4], production conditions [5,6], substrates used [7,8], feeding regimes [9,10], processing [11,12], and final products [13,14,15].

*Hermetia illucens* can be fed with a wide range of substrates, whether they are agricultural production wastes, processed food, meat wastes, or even faecal matter, depending on the final use of the insect, which may be the larva itself (fresh, dehydrated, or frozen), flour, oil, compost, or chitin, for the most diverse purposes. This means that the production of this insect, at any level, can be a key element in a circular economy model; nevertheless, there are some reservations in their use for food and feed purposes that, for instance, in European Union, restrict the authorized feeding wastes to vegetables and unprocessed former foodstuff (such as dairy and eggs), avoiding high-risk substrates, such as manure and former foodstuffs containing meat or fish [16,17]. Due to the fact that a huge percentage of waste production and food losses occur in the production chain before reaching the market and consumers, and vegetables and fruits can make up 40–50% of the total amount [18], using vegetable wastes as feed for insects can be a great mechanism to increase sustainability along the value chain. Although insects can be key agents to achieve the United Nations Sustainability Goals (SDG), there are some concerns regarding this new production model. For instance, in what refers to the assimilation by feed markets, since the price of insect-based protein is still an obstacle, being relatively higher compared with commonly used protein sources, such as fishmeal and soymeal [19]. On the other hand, the environmental burden related with insect rearing is not as low as initially expected [20]. One of the major advantages of insect bioconversion of food into insect biomass is due to the fact that, since they are poikilothermic, meaning that their metabolism is not used to maintain their body temperature, all nutrients are used for biomass development; however, it implies the need to use high temperatures and, consequently, a higher energy consumption in more northern latitudes [21]. In addition to energy consumption, feeding substrates may be a constraint at industrial scales, since higher quality substrates lead to greater yields of insect production, but also can lead to higher environmental burdens related to feed production [20]. The best approach should be the optimization of the production model in terms of energetic efficiency, either by energy self-consumption or, simply by lowering the operation temperatures and using low quality substrates, such as food waste or agricultural by-products.

Although many advances have been made regarding BSFL production, there are still a knowledge gap regarding its growth behaviour when fed with specific substrates. Even if BSFL can be successfully reared in generic vegetable residues, preliminary studies to this trial (data not shown) point to substantial differences in BSFL growth patterns in certain fruits and/or vegetables, which can indicate a poor nutrient balance or the existence of some inhibiting growth factors. As a primary food source, plants evolved and developed several mechanisms to face the attacks of herbivores, including the production of chemical substances called secondary metabolites [22]. Among these, some have recognized insecticidal or pernicious properties over several insect families, such as tannins, alkaloids, terpenoids, flavonoids, cyanogenic glycosides, saponins, phenolics, and sulphur-containing compounds [23]. Some insect species are able to self-select their diet, which means that they are able to regulate the intake of nutrients [24], so it is possible that, when in the presence of substrate mixtures, insects avoid those that may have antinutritional properties and choose substrates in which they can have better development performances.

The apple is among the most consumed fruits, with a consequently higher food waste and the potential to be used as substrate for BSF larvae production. This fruit is rich in several flavonoids, among other potential harmful phytochemicals to insects, such as anthocyanins, catechin, and epicatechin, present in peels and flesh [25], but also contains high levels of cyanogenic glycosides in the seeds [26]. Flavonoids such as quercetin and kaempferol are also present in red cabbage as well as chlorogenic acids and lignans [27,28,29]. Red onion is another vegetable with antioxidant properties containing anthocyanins, flavonols, saponins, and chlorogenic acids [30,31]. At lower levels than apples, pumpkins contain cyanide in their seeds among other polyphenols, such as chlorogenic acids, lignans, and flavones [28,32,33]. Grape pomace is rich in tannins and grape seeds are good sources of phenolic acids, catechin, and epicatechin, as well as anthocyanins [34,35]. Spinach is also one of the richest vegetables in secondary metabolites, containing high levels of diterpenoids, oxalate, lignin, tannins, and saponins [29,36].

In addition to the substrate qualitative perspective, the use of different feeding strategies, batch or continuous systems, is also a factor to be considered regarding larvae production efficiency. BSFL are known to cease or reduce feeding under unfavourable conditions [37], which may affect the yield of BSFL in industrial rearing. It is possible that, when larvae are fed at once during their life cycle, in a batch model, the substrate rots due to microorganism action and nutrients become less available to larvae or even that larvae have a preference for a fresh substrate [9,38]. On the other hand, a continuous system, in which new substrate is supplied whenever the substrate appears to be consumed every two or three days, may lead to moments, between substrate feeding events, where there is a lack of nutrients available for larvae development [10].

To the authors’ knowledge, there are few studies that have focused on larval performances fed with organic mono-streams with potential anti-nutritional properties to black soldier fly larvae. From an industrial perspective, it is important to have as much knowledge as possible about the factors that interfere in the production yield, whether they are substrate typology, physical conditions, or process tuning. Vegetable and fruit wastes have, generally, a high water content so it may require dehydration before being supplied to larvae to avoid a reduction in the production yield. 

The objective of this study is to assess the effects of suboptimal conditions in the development performance and conversion ratios of BSFL fed with different single vegetable diets with specific secondary metabolite content, under different temperatures and substrate moistures, in a continuous feeding regime.

## 2. Materials and Methods

### 2.1. Larvae Rearing and Sample Collection

The trial was conducted in the Coimbra Agriculture School from the Coimbra Polytechnic Institute and the larvae used were supplied by the local pilot rearing unit. This colony was maintained under controlled conditions (14L:10D, at 50 ± 5% RH and 27 ± 2 °C) and the larvae were fed with raw fruit and vegetable wastes from the campus’ canteen. Eggs with less than 24 h were collected in egg traps and were incubated in an environmental chamber (model STF-F 52-Lt; Falc co., Treviglio, Italy), in plastic boxes with wheat bran moisturized with water (70%), at 27 ± 2 °C and 50 ± 5% RH until the feeding trials.

### 2.2. Substrate Preparation

The substrates used in the trials were supplied by local producers and were chosen based on their typical composition in anti-nutritional compounds for insects, as well as their availability as commonly used substrates [39]. In addition to wheat bran (CONTROL), six plant-based substrates were used: apple (A), red cabbage (RC), red onion (RO), pumpkin (P), grape pomace (Pom), and spinach (S).

Since substrate moisture is known to influence BSFL performance, two substrate moisture conditions were tested: an optimal substrate moisture (70% SM) and a natural substrate moisture (NAT SM) of plant-based substrates [40]. All substates were dehydrated for the optimal moisture content trial, except for grape pomace that, due to its low moisture content (49.5%), had to be moisturized with distilled water. The control substrate (wheat bran) had a low moisture content at natural conditions (9.9%), for which it was moisturized with distilled water to achieve both optimal moisture and to simulate a high vegetable natural moisture (90%). The plant-based substrates were chopped and frozen at the beginning of the trial and unfrozen at ambient temperature before feeding. The approximate nutritional composition for trial substrates was estimated from values reported in the literature and is shown in Table 1.

### 2.3. Experimental Design and Larval Performance

At the beginning of the trial, a total of 5–6-day-old 100 larvae per replicate (5 replicates per substrate) were weighted and assigned to 120 mL plastic containers covered with a net lid, containing 3 ± 0.1 g of substrate. Each substrate replicate was then placed in climatic chambers (60–70% RH; 0L:24D photoperiod) with different temperature conditions (20, 25, and 30 °C) and substrate moisture (SM) (70% and NAT). The conditions of different treatments are summarized in Table 2.

Every 2–3 days, 20 larvae were randomly collected, cleaned with a paper towel, and weighted. After weighting, these larvae were reintroduced in the container and more substrate was added whenever necessary. 

Whenever a larva reached the prepupa stage, it was removed from the container and weighted. It was decided to end the trials whenever 50% of individuals reached the prepupal stage, since the duration of the larval instar in some of the substrates largely exceeded the one from the control treatment. The larvae and prepupae were then separated from the substrate, cleaned, and weighted. The substrates were then dehydrated at 105 °C for 24 h until constant weight and their weight and moisture content registered [43].

For the evaluation of BSFL performance under the studied conditions, several parameters were calculated, per replicate, at the end of the trial: survival rate (SUR, Equation (1)), growth rate (GR, Equation (2)), and fresh weight of larvae and prepupae.

(1) $$\mathrm{SUR}\;(\%)=\frac{\mathrm{Number}\;\mathrm{of}\;\mathrm{larvae}\;\mathrm{harvested}\;}{\mathrm{Initial}\;\mathrm{number}\;\mathrm{of}\;\mathrm{larvae}\;}\times 100$$

(2) $$\mathrm{GR}\;\left(g/d\right)=\frac{\mathrm{Biomass}\;\mathrm{weight}\;\mathrm{gain}\;}{\mathrm{Trial}\;\mathrm{duration}\;}$$

### 2.4. Waste Reduction and Conversion Efficiency

Bioconversion efficiency to reduce organic matter by BSFL was assessed by the determination of 4 of the most used parameters for both waste reduction efficiency and feed to body mass conversion. Waste degradation was estimated through waste reduction index (WRI, Equation (3)), while for bioconversion performance, the feed conversion rate (FCR, Equation (4)), bioconversion rate (BR, Equation (5)), and efficiency of conversion of digested feed (ECD, Equation (6)) were calculated, as described in previous studies [43,44].

(3) $$\mathrm{WRI}=\frac{\frac{\mathrm{Substrate}\;\mathrm{added}-\mathrm{Residual}\;\mathrm{Substrate}}{\mathrm{Substrate}\;\mathrm{added}}}{\mathrm{Trial}\;\mathrm{duration}\;}\times 100$$

(4) $$\mathrm{FCR}=\frac{\mathrm{Substrate}\;\mathrm{added}-\mathrm{Residual}\;\mathrm{Substrate}}{\mathrm{Final}\;\mathrm{biomass}\;}$$

(5) $$\mathrm{BR}\;(\%)=\frac{\mathrm{Final}\;\mathrm{biomass}}{\mathrm{Substrate}\;\mathrm{added}}\times 100$$

(6) $$\mathrm{ECD}\;\left(\%\right)=\frac{\mathrm{Biomass}\;\mathrm{gain}}{\mathrm{Subtrate}\;\mathrm{added}-\mathrm{Residual}\;\mathrm{substrate}}$$

### 2.5. Statistical Analysis

The statistical differences between treatments were validated by a permutational multivariate analysis of variance (PERMANOVA; 9999 permutations) using software PRIMER v.7. After PERMANOVA, pairwise comparisons among levels of factors were performed to assess significant differences between them, using pseudo-t and *p*-values by permutation [45]. The relationship and variability associated with mean values of treatments and calculated parameters was assessed with a principal components analysis (PCA), using the software PAST v.4.07b.

The inexistence of living larvae at the end of the trial in grape pomace replicates at 20 and 30 °C NAT, made a global statistical analysis unfeasible for BR, FCR, and ECD parameters. Consequently, the analysis of variance was performed at two degrees: a 3-way PERMANOVA for remaining substrates at all conditions, and a 2-way PERMANOVA for all substrates at 70% SM. Since the survival rate was the parameter in which the absence of value (0%) had a statistical relevance, it was the exception, and the analysis reflected all replicates at all conditions.

All statistical analysis results may be seen for further details in the Supplementary Material.

## 3. Results

### 3.1. Larval Performance

**Figure 1:**
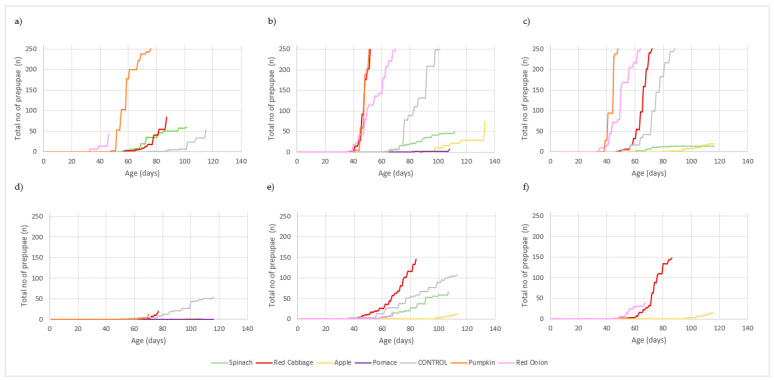
Black soldier fly prepupal yield (sum of total prepupae of all replicates over trial duration) —(**a**) 20 °C 70% SM; (**b**) 25 °C 70% SM; (**c**) 30 °C 70% SM; (**d**) 20 °C NAT SM; (**e**) 25 °C NAT SM; (**f**) 30 °C NAT SM.

The evolution of the total number of prepupae for each combination of substrate/temperature/humidity over the trial duration is presented in Figure 1. 

The best larval performance was achieved in substrates at 70% SM, when the larvae were fed with pumpkin. In this treatment, 50% larvae moulted into prepupae earlier than in other treatments, taking 51 and 48 days at 25 °C and 30 °C, respectively. Larvae fed with red onion moulted into prepupae in 69 (25 °C) and 64 days (30 °C), and larvae fed with red cabbage took 52 (25 °C) and 73 days (30 °C). The control replicates took 99 (25 °C) and 85 days (30 °C), the longest observed larval instar duration until 50% moulted. Under the conditions of 20 °C and 70% SM, only larvae fed with the pumpkin substrate achieved 50% of prepupae.

Higher temperatures also contribute for the faster development of BSFL. It is clear that all substrates obtained a better yield at 30 °C, with the exception of apple and spinach where larvae developed faster at 25 °C for 70% SM conditions. At NAT SM conditions, the temperature of 25 °C led to a faster development of larvae until prepupae, even though, as referred before, none of the treatments achieved 50% of prepupae.

**Figure 2:**
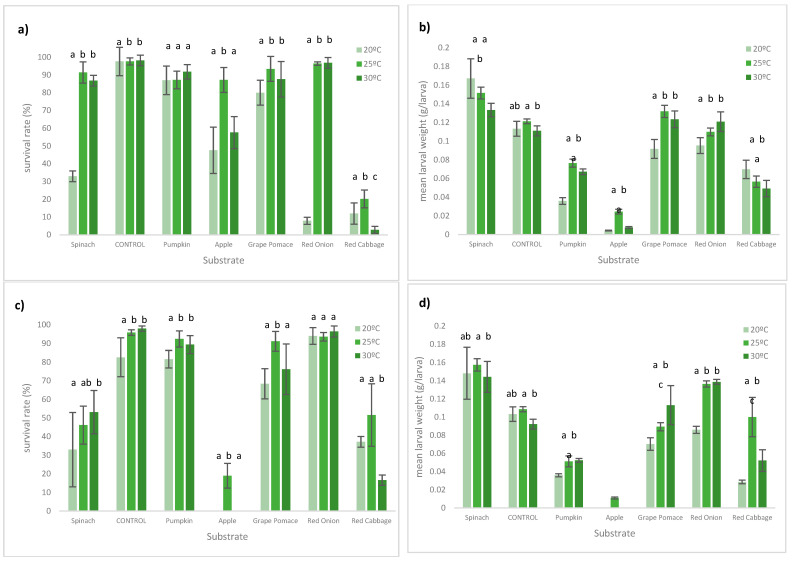
BSFL performances under different temperatures at the end of the trial. (**a**) Survival rates (%) at 70%SM; (**b**) average final biomass per larvae at 70%SM (larvae + prepupae ± SD); (**c**) survival rates (%) at NAT; (**d**) average final biomass per larvae at NAT (larvae + prepupae ± SD). Significant differences between the tested substrates (*p* ≤ 0.05) are represented by different letters. Grape pomace weight at NAT SM was not included in the analysis.

The survival rates and biomass weight of larvae and prepupae under different substrates and conditions are shown in Figure 2 (for further details, see Supplementary Material Table S1). There were significant differences between survival rates (*p* = 0.0001) and biomass weight (*p* = 0.0001) among all tested substrates and conditions. 

Overall, the highest survival rates were achieved using pumpkin as a substrate, reaching 98.2%, at 30 °C, 70% SM. The grape pomace had the lowest survival rate of all substrates at natural conditions, with no alive larvae at 20 and 30 °C and almost none at 25° at NAT conditions. Low survival rates were also observed in larvae fed with spinach.

The results for biomass larval weight (larvae + prepupae) showed heavier larvae in the control samples, namely at 20 °C 70% SM and 25 °C NAT, respectively, for 0.167 g/larva and 0.158 g/larva. These heavier larvae, when analysed in combination with the growth rate (*p* = 0.001) or survival rate (*p* = 0.001), lose some consistency as they were obtained at the expense of a longer cycle duration (Table S1). In fact, these two higher values of biomass weight were observed with survival rates of 33% and 46%, respectively. Pumpkin and red onion achieved a heavier biomass in samples with lower moisture content, while red cabbage presented the opposite behaviour. Nevertheless, all these three substrates had higher growth rates at a 70% moisture content. Despite having reasonably high levels of survivance (81.6–92.4%), apple had extremely low growth rates in all studied conditions (0.259–0.564 mg/d). 

### 3.2. Waste Reduction and Conversion Efficiency

The results for biomass conversion parameters calculated are shown in Table 3. All substrates had significant effects on the calculated waste reduction and conversion efficiency parameters calculated for both assessed groups: all substrates at 70% SM and all conditions without grape pomace (see supplementary material Tables S2–S4). Temperature had no significant effect in the feed conversion ratio (FCR) (*p* = 0.1016) when all substrates were considered at 70% SM. In the statistical analysis of all temperature and moisture conditions of substrates excluding grape pomace, the interaction of temperature and moisture had no significant effect over the results of the bioconversion ratio (BR) (*p* = 0.9224) and FCR (*p* = 0.4926) nor on the interaction between all factors over the digested feed ratio ECD results (*p* = 0.0761).

The substrates that achieved better larval development resulted in a higher waste reduction index. In fact, larvae reared in pumpkin at 30 °C 70% SM achieved higher values of the waste reduction index (WRI) (1.75), while grape pomace at 25 °C NAT had the lowest value (0.4). The lower WRI values for the grape pomace were expected since the survival rates were low too. Spinach presented a better WRI than apple for all replicates at 70% SM and at 25 °C NAT. 

Regarding conversion efficiency, which is an indicator of the capability of converting feed into biomass, it is important to consider the existence of two types of opposite signal parameters. BR and ECD indicate the weight of biomass achieved for an amount of substrate added (total or digested), while the FCR is the amount of feed that is needed to reach one unit of biomass. In this way, high values of BR and ECD are good indicators of feed conversion efficiency and high values of FCR indicate a poor assimilation of the substrate.

A lower larval development represents high FCR values, either by having small larvae at the end of the trial or due to the low number of surviving larvae. If the larval weight of the grape pomace samples explains the high values of FCR for this substrate, the poor performance on spinach in this indicator results from the observed high mortality. The three substrates with better results in FCR are pumpkin (FCR = 4 at 25 °C for both moisture contents), red onion (FCR = 4.2 at 25 °C; 70% SM), and red cabbage (FCR = 4.6 at 25 °C; 70% SM).

Regarding the other two calculated bioconversion indicators, in general, samples at 70% SM showed better conversion results than samples at NAT conditions. The substrate with the best value of BR was red cabbage (BR = 13.6% at 25 °C; 70% SM), which means that, from the studied vegetables, this was the one that BSFL reduced more efficiently to produce biomass. Nevertheless, when considering the substrate residues and therefore the larval substrate assimilation, pumpkin (at 25 °C for both moisture content conditions) was the substrate where BSFL was more efficient in its conversion to biomass (ECD = 25%).

The control samples had better waste reduction and conversion efficiency results than the three worst substrates (apple, grape pomace, and spinach) and worse than the three best substrates (pumpkin, red cabbage, and red onion) in all calculated parameters.

The results regarding the ingested feed are just informative since this was a continuous process that was strongly dependent on the nature of the tested substrate; the comparison is only between the conversion ratios when larvae were feed with the different substrates under the different tested conditions (temperature and moisture).

### 3.3. Principal Component Analysis

**Figure 3:**
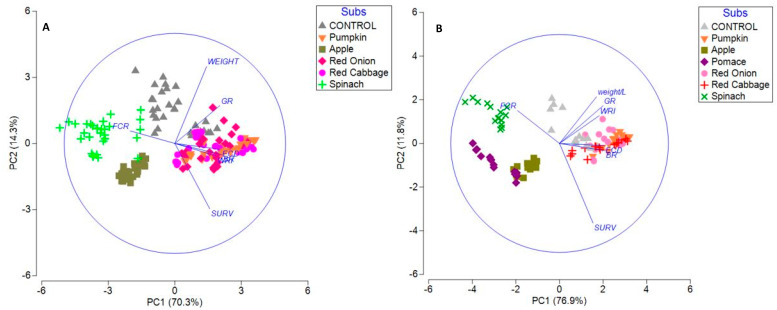
Principal component analysis loading plots for black soldier fly performance and conversion parameters calculated (WRI—waste reduction index; FCR—fed conversion ratio; BR—bioconversion ratio; ECD—efficiency of conversion of digested feed; GR—growth rate). (**A**)—All conditions (without grape pomace); (**B**)—70% SM (all substrates).

Two PCA (Figure 3) were performed for all substrates at 70% SM and for all temperatures and moisture conditions with and without the grape pomace results, since it was not possible to calculate several parameters (FCR, ECD, and BR) due to the 0% survival rate observed in this substrate.

The distribution of variability is mainly driven by two principal components that account for 81.2% (Figure 3A) and 84.6% (Figure 3B) of the total variance. From the PCA it is possible to observe that the results are grouped by substrates more than by temperature or moisture content, which indicates that, for this trial, the factor substrate has more influence than the other two factors. Pumpkin, red cabbage, and red onion were the substrates that achieved the highest values of performance and conversion, while grape pomace, apple, and spinach achieved the worst. The Control samples were roughly located in the centre of the plot in both PCA, but this was the only treatment in which the effects of temperature and moisture were evident.

## 4. Discussion

Currently, one third of all food produced globally is thrown away each year. This represents a huge loss of nutrients and a major cause of environmental concern [18]. The commitment of the EU with the UN SDG aims to halve food waste at the consumer and retail levels by 2030 and reduce food losses along the production and supply chains (mainly SDG 12.3).

Detritivorous insects, such BSF, may be active agents in the recovery of these lost nutrients, transforming organic residues into valuable nutrients.

It is known that *Hermetia illucens* may reduce their metabolism to face unfavourable conditions, such as feed scarcity [10,46], but the addition of substrate in suboptimal quantities aimed to: (a) avoid dietary self-selection and (b) induce the consumption of substrate elements that larvae would reject in abundance conditions, thus maximizing the potential effect of any anti-nutritional property.

Our results show that BSF vegetables waste bioconversion was non-linear varying with the type of vegetable. For instance, regarding pupation, even though there were differences between the substrates, the duration of these feeding trials was considerably longer in comparison with similar studies with vegetable and fruit wastes [47,48]. This was not surprising since the amount of substrate fed to BSFL was lower than the optimal amount reported for the larval development [43]. As already observed, BSFL developed faster and achieved higher yields (number of prepupae) at 70% SM, at all tested temperatures, in comparison with NAT SM. Similar results were observed by [49] et al. and Cammack and Tomberlin [50], which established 70% as an optimal substrate moisture content. The containers used in our trials did not have any drainage mechanism; for this reason, water tended to accumulate over the substrate, which negatively influenced larval feeding. The lower development duration observed for replicates at 30 °C than at 20 or 25 °C was in line to those observed by Chia et al. [51], who reported lower larval development times at equal temperature conditions. The influence of temperature is an important issue when it is necessary to maintain high levels of productivity, at an industrial scale, especially in higher latitudinal regions, which implies higher energy consumption for heat.

The high levels of mortality in larvae fed with grape pomace at NAT SM conditions may be explained by the intrinsic disadvantageous characteristics of this substrate for BSFL performance, mostly related with its low water content, high proportion of woody material, recalcitrant compounds, and the presence of potentially harmful secondary metabolites that can potentially hamper larvae consumption, despite presenting reasonable protein levels [34,35]. The high proportion of woody materials may also explain the higher survival rates observed at 70% SM in comparison with NAT. The higher water content at this condition prevented the desiccation of BSFL, even if it did not result in a good biomass weight, which probably indicates a low quality of feed material. This trial was conducted at substrate suboptimal proportions, which means that the high substrate surface per volume could originate more water loss. It is possible that higher proportions of grape pomace could avoid the desiccation of larvae, or even lead to more assimilable substrate (grape pulp and peels). 

The low survival rates observed in the larvae fed with spinach can be a consequence of its high content in polyphenols and, consequently, anti-nutritional or even potential insecticidal effects, which can indicate that it is a vegetable to avoid when rearing BSFL [29,36]. Another hypothesis is that, since spinach is one of the most contaminated crops with pesticide residues [52], the spinach samples used in this trial could have contained pesticide residues, which can explain the observed mortality. Additionally, some pesticide content can increase after dehydration to achieve 70% SM, explaining the additional mortality at this condition [53]. The temperature of 30 °C, apparently, had a higher effect on the mortality of larvae fed with spinach, observed in the two studied moisture contents, which may indicate the increase in the insecticide potential of this vegetable over BSFL. The correlation between temperature and pesticides was also observed in other insect orders, such as Coleoptera [54] and Ephemeroptera [55].

The heavier larvae biomass in the control replicates, where survival rates achieved poor results, was in accordance to what was reported by Lalander et al. [56], who observed that a higher water content increased larval weight at low survival rates and decreased larval weight at higher survival rates. It is possible too, especially in suboptimal substrate conditions, that the mortality led to more substrate being available for the remaining larvae or even cannibalism occurrence, as shown by Nguyen [57]; however, this was not reflected in a normal cycle duration in this study. Broeckx et al. [58] reported less biomass weight despite good survival rates in larvae fed with apple and advanced the low protein content of apples as a possible cause, based on Liland et al.’s [59] observations using seaweeds. This author also pointed for the fact that apples are rich in crude fibre content and cellulose, which may interfere with digestive processes. However, both Meneguz et al. [60] and Danieli et al. [61] reported normal results with high fibre content substrates. Scala et al. [62] observed better performances with apple, in industrial quantities, but there is the possibility that the placement of a dry Gainsville diet around the perimeter of the experimental diets offered additional feed to larvae. In fact, it cannot be excluded that the Gainsville diet had been moistened in the boundaries by the experimental diet. Given our observed results, it is possible that the high level of tannins in apples act as larval development deterrents, but in amounts lower that those that induce mortality. 

Apparently, there was not any effect regarding the protein content of the substrates per se on BSFL performance, since the two substrates with a higher protein content, grape pomace at NAT SM and spinach at 70% SM, display poor results in all the studied parameters. Barragan-Fonseca et al. [63] observed that the higher the protein and carbohydrate (P + C) content values, the better were the results in terms of larvae development, but, in this trial, wheat and apple, despite their higher levels of P + C, did not achieve similar results. The poor performances observed in the larvae fed with apple in our trial may indicate that other nutritional characteristics in addition to protein and carbohydrates may influence larval development.

In general, the results of the waste reduction index (WRI) (0.40–1.54) are lower than those observed in previous studies [9,43,60,63], which used several substrates, including fruit wastes (3.59–4.85), canteen wastes (4.3), vegetable wastes (3.2), or chicken feed (1.1–3.8). These lower WRI values were expected due to the slower larvae development that was incorporated in this parameter calculation. The observation of better WRI results in spinach in comparison to apple may reinforce the hypothesis that the higher consumption of spinach had insecticidal effects over the larvae, while the larvae fed with apple may be continuously feeding but without growing efficiently.

The feed conversion ratio is an indicator that translates the amount of feed needed to achieve biomass weight without attention to life cycle duration; thus, the best trial results for this parameter (pumpkin, red onion, and red cabbage) were expected to be similar to those of other similar studies (FCR = 4.5–15.3), despite the longer periods of larval development observed [10,38,64].

The bioconversion rate results are in line with those observed by Lalander et al. [56] in what concerns the higher conversion levels in substrates with 70% SM, which is near the optimal moisture content for BSFL development. The bioconversion values results achieved in this study are in accordance with what was observed in previous studies where vegetables and fruits were used as substrate when calculated in a substrate total basis (BR = 5.35–29%), but were higher considering the substrate that is effectively digested (ECD = 5–7%) [10,60,64].

The vegetables and fruits tested in this trial are some of the richest in what concerns total phenolic content [65,66]. However, each one had its own qualitative profile and it is possible that the three substrates with worst performances—apple, grape pomace, and spinach—contain anti-nutritional phenolic properties. In fact, spinach has been reported to have insecticidal effect in *Drosophila melanogaster* [67]. Grapes are rich in phenolic compounds that are mainly present in the seeds, skins, and stems [68], which correspond to the substrates tested in this trial. This phenolic content may be the reason for the poor results observed in this trial, even though the high level of woody material and lack of material capable of being assimilated must be considered. Although red onion is also a vegetable rich in polyphenols, these are found mainly in the outer skin, which could have been possibly avoided by larvae.

## 5. Conclusions

BSFL performance depends on different factors, including the feeding substrate which is used. This influence concerns its nature, availability or moisture content, and environmental conditions, such as temperature. In this work, BSFL performance and bioconversion parameters at suboptimal conditions were obtained and these results contribute to the optimization of mass production scale operations.

It was possible to corroborate that, under suboptimal feeding conditions, a substrate moisture content of 70% was more suitable for BSFL development than the original moisture content for almost all rearing conditions and parameters assessed. Temperatures ranging from 25 to 30 °C were also confirmed as the most suitable for BSFL development. 

The nature of the substrates used had more impactful effects than the temperature or moisture. Feeding larvae with pumpkin, red cabbage, and red onion resulted in higher conversions by BSFL, whereas apple, spinach, and grape pomace turned out to be poor substrates for *Hermetia illucens* rearing. While apple led to a poorer and slower development performance, spinach and grape pomace showed higher mortality rates, indicating that there were different specific effects depending on the vegetable composition, which may be related to anti-nutritional aspects. 

The results obtained in this work provide information for a better selection of substrates on an industrial scale to obtain higher yields in a shorter production time, increasing the efficiency of BSFL industrial production. When operating under a circular economy system, the choice of the wastes or vegetable supplier is a central factor to consider, especially if this is a mono-stream by-product.

Future research is needed to assess if the obtained results suffer changes when supplying optimal substrate amounts and the nutritional aspects of larvae reared in these substrates, in particular the influence of polyphenols.

## Figures and Tables

**Table 1:** Estimated nutritional composition of the plant-based substrates used in the trial under natural conditions (NAT SM) and under optimal substrate moisture conditions (70% SM) [41,42].

		Wheat Bran	Apple	Grape Pomace	Pumpkin	Red Cabbage	Red Onion	Spinach
Plant-based substrate composition (g/100 g wet basis)	Moisture	9.9	81.2	49.5	86.47	88.9	83.5	92
	Protein	15.1	0.3	5.8	1	1.4	0.9	2.9
	Carbohydrate	61.6	13.8	13.0	10.5	7.4	9.9	2.6
	Fat	2.73	0.2	2.8	0.1	0.2	0.1	0.6
	Fibre	10.4	2.4	12.2	2	2.1	2.2	1.6
	**TRIAL**							
NAT SM (g/100 g wet basis)	Protein	1.7	0.3	5.8	1	1.4	0.9	2.9
	Carbohydrate	6.8	13.8	13.0	10.5	7.4	9.9	2.6
	Fat	0.3	0.2	2.8	0.1	0.2	0.1	0.6
	Fibre	1.2	2.4	12.2	2	2.1	2.2	1.6
	Moisture	90	81.2	49.5	86.47	88.9	83.5	92
70% SM (g/100 g wet basis)	Protein	5	0.5	3.4	2.2	3.8	1.7	10.8
	Carbohydrate	20.5	22	7.7	23.2	20.1	18	9.7
	Fat	0.9	0.3	1.7	0.2	0.5	0.2	2.2
	Fibre	3.5	3.8	7.3	4.4	5.7	4.4	6.0

**Table 2:** Experimental design and codification of groups of samples for the trial.

Temp. (°C)	Substrate Moisture (%)	Control	Apple	Grape Pomace	Pumpkin	Red Cabbage	Red Onion	Spinach
20	70	C7020	A7020	Pom7020	P7020	RC7020	RO7020	S7020
25		C7025	A7025	Pom7025	P7025	RC7025	RO7025	S7025
30		C7030	A7030	Pom7030	P7030	RC7030	RO7030	S7030
20	NAT	CN20	AN20	PomN20	PN20	RCN20	RON20	SN20
25		CN25	AN25	PomN25	PN25	RCN25	RON25	SN25
30		CN30	AN30	PomN30	PN30	RCN30	RON25	SN30

**Table 3:** Comparison of black soldier fly conversion parameters (mean ± SD) fed with the tested substrates under different tested conditions (temperature and moisture); WRI—waste reduction index (dry matter); FCR—feed conversion ratio; BR—bioconversion ratio; ECD—efficiency of conversion of digested feed.

Substrate	Temp. (°C)	SM (%H_2_O)	Feed ^1^ (mg/larva.d)	WRI ^2^	FCR ^2^	BR ^2^ (%)	ECD ^2^ (%)
Control	20	70	2.890 ± 0.003	0.54 ^a^ ± 0.03	12.0 ^a^ ± 1.7	5.1 ^a^ ± 0.9	8.5 ^a^ ± 1.3
Pumpkin			4.537 ± 0.341	1.28 ^b^ ± 0.50	5.0 ^b^ ± 0.4	11.8 ^b^ ± 0.9	20.0 ^b^ ± 1.5
Apple			2.281 ± 0.012	0.47 ^a^ ± 0.03	23.9 ^c^ ± 2.2	3.1 ^a^ ± 0.4	4.2 ^c^ ± 0.4
Grape Pomace			1.471 ± 0.004	0.41 ^c^ ± 0.01	225.8 ^d^ ± 40.9	0.4 ^c^ ± 0.1	0.5 ^d^ ± 0.1
Red Onion			2.437 ± 0.007	1.18 ^b^ ± 0.01	5.2 ^b^ ± 0.5	12.6 ^b^ ± 0.9	19.3 ^e^ ± 1.6
Red Cabbage			2.497 ± 0.003	0.79 ^d^ ± 0.05	6.1 ^e^ ± 0.6	11.3 ^b^ ± 1.3	16.5 ^ae^ ± 1.7
Spinach			2.047 ± 0.137	0.67 ^a^ ± 0.11	71.9 ^f^ ± 7.0	1.3 ^d^ ± 0.1	1.4 ^f^ ± 0.1
Control	25		4.709 ± 0.051	0.73 ^a^ ± 0.01	5.6 ^a^ ± 0.3	9.3 ^a^ ± 0.6	18.0 ^a^ ± 1.0
Pumpkin			6.904 ± 0.009	1.56 ^b^ ± 0.02	4.0 ^b^ ± 0.1	11.2 ^b^ ± 0.4	25.0 ^b^ ± 0.6
Apple			3.797 ± 0.002	0.57 ^c^ ± 0.01	16.0 ^c^ ± 0.6	4.0 ^c^ ± 0.1	6.2 ^c^ ± 0.2
Grape Pomace			1.709 ± 0.007	0.50 ^d^ ± 0.01	24.7 ^d^ ± 1.2	3.4 ^d^ ± 0.2	4.1 ^d^ ± 0.2
Red Onion			4.742 ± 0.033	1.36 ^b^ ± 0.06	4.2 ^e^ ± 0.4	12.5 ^b^ ± 0.6	24.0 ^e^ ± 2.3
Red Cabbage			4.820 ± 0.168	1.65 ^b^ ± 0.06	4.6 ^a^ ± 0.3	13.6 ^b^ ± 0.6	21.8 ^a^ ± 1.3
Spinach			2.835 ± 0.152	0.68 ^a^ ± 0.06	60.0 ^d^ ± 10.9	1.2 ^c^ ± 0.2	1.7 ^d^ ± 0.3
Control	30		5.322 ± 0.055	0.96 ^a^ ± 0.05	9.9 ^a^ ± 0.6	8.2 ^a^ ± 0.4	10.1 ^a^ ± 0.7
Pumpkin			7.090 ± 0.066	1.75 ^b^ ± 0.04	5.7 ^b^ ± 0.2	11.0 ^b^ ± 0.4	17.5 ^b^ ± 0.8
Apple			4.023 ± 0.004	0.55 ^c^ ± 0.01	22.8 ^c^ ± 0.7	3.5 ^c^ ± 0.1	4.4 ^c^ ± 0.1
Grape Pomace			2.021 ± 0.009	0.45 ^d^ ± 0.02	44.0 ^d^ ± 6.4	0.6 ^d^ ± 0.1	0.7 ^d^ ± 0.1
Red Onion			5.368 ± 0.084	1.54 ^b^ ± 0.04	7.0 ^b^ ± 0.5	10.9 ^b^ ± 0.8	14.4 ^b^ ± 1.1
Red Cabbage			4.270 ± 0.110	1.21 ^e^ ± 0.03	6.3 ^b^ ± 0.5	12.6 ^b^ ± 1.4	16.0 ^b^ ± 1.5
Spinach			2.990 ± 0.166	0.78 ^ac^ ± 0.07	960.0 ^e^ ± 763.6	0.2 ^d^ ± 0.1	0.2 ^d^ ± 0.2
Control	20	NAT	0.731 ± 0.086	0.51 ^a^ ± 0.03	16.0 ^a^ ± 14.4	1.4 ^a^ ± 0.2	9.1 ^a^ ± 4.4
Pumpkin			2.006 ± 0.002	1.28 ^b^ ± 0.01	6.10 ^b^ ± 0.4	6.9 ^b^ ± 0.0	16.6 ^b^ ± 1.1
Apple			1.405 ± 0.003	0.55 ^c^ ± 0.01	26.7 ^c^ ± 1.7	3.1 ^c^ ± 0.0	3.8 ^c^ ± 0.2
Grape Pomace			2.595 ± 0.266	0.46 ± 0.05	-	-	-
Red Onion			1.454 ± 0.002	1.46 ^d^ ± 0.02	6.8 ^d^ ± 0.4	8.8 ^d^ ± 0.3	8.7 ^a^ ± 0.3
Red Cabbage			1.388 ± 0.004	0.96 ^e^ ± 0.02	8.9 ^a^ ± 0.2	7.7 ^e^ ± 0.2	7.8 ^a^ ± 0.2
Spinach			0.624 ± 0.004	0.49 ^a^ ± 0.03	68.9 ^e^ ± 8.8	1.2 ^f^ ± 0.0	1.5 ^d^ ± 0.2
Control	25		1.285 ± 0.085	0.57 ^a^ ± 0.01	10.0 ^a^ ± 2.4	5.9 ^a^ ± 0.4	10.4 ^a^ ± 2.1
Pumpkin			2.359 ± 0.001	1.31 ^b^ ± 0.01	4.0 ^b^ ± 0.2	7.7 ^b^ ± 0.0	25.0 ^b^ ± 1.0
Apple			2.265 ± 0.010	0.53 ^c^ ± 0.01	17.7 ^c^ ± 2.1	2.6 ^c^ ± 0.0	5.7 ^c^ ± 0.7
Grape Pomace			2.591 ± 0.006	0.40 ± 0.01	203.6 ± 68.6	0.4 ± 0.0	0.5 ± 0.2
Red Onion			2.419 ± 0.003	1.52 ^d^ ± 0.00	4.8 ^d^ ± 0.2	9.0 ^d^ ± 0.0	21.0 ^d^ ± 0.9
Red Cabbage			2.212 ± 0.003	1.07 ^e^ ± 0.00	8.6 ^a^ ± 0.2	7.9 ^e^ ± 0.0	11.6 ^a^ ± 0.3
Spinach			1.057 ± 0.002	0.65 ^f^ ± 0.03	21.9 ^e^ ± 2.7	3.0 ^f^ ± 0.0	4.6 ^e^ ± 0.5
Control	30		1.599 ± 0.001	0.61 ^a^ ± 0.02	19.9 ^a^ ± 4.8	4.3 ^a^ ± 0.0	5.2 ^a^ ± 1.0
Pumpkin			2.399 ± 0.003	1.26 ^b^ ± 0.02	11.1 ^b^ ± 0.8	7.0 ^b^ ± 0.0	9.1 ^b^ ± 0.6
Apple			2.652 ± 0.002	0.59 ^a^ ± 0.01	31.0 ^c^ ± 1.3	2.5 ^c^ ± 0.0	3.2 ^c^ ± 0.1
Grape Pomace			3.344 ± 0.239	0.57 ± 0.03	-	-	-
Red Onion			2.665 ± 0.005	1.49 ^c^ ± 0.02	9.0 ^d^ ± 0.5	8.4 ^d^ ± 0.0	0.111 ^d^ ± 0.6
Red Cabbage			2.401 ± 0.003	1.15 ^d^ ± 0.01	11.9 ^b^ ± 0.3	7.6 ^e^ ± 0.0	0.084 ^b^ ± 0.2
Spinach			1.069 ± 0.002	0.51 ^e^ ± 0.02	147 ^e^ ± 44.3	0.7 ^f^ ± 0.0	0.7 ^e^ ± 0.2

^1^ Dry matter basis. ^2^ Means in a column within groups of TempxSM, which are followed by the same letter, are not significantly different (*p* ≤ 0.05).

## Data Availability

All relevant data are within the paper.

## Supplementary Materials

The following supporting information can be downloaded at: https://www.mdpi.com/article/10.3390/insects13070639/s1. Table S1: Black soldier fly development parameters; Table S2: Three-way PERMANOVA analysis and pair-wise tests for survival results; Table S3: Two-way PERMANOVA analysis and pair-wise tests for development and bioconversion variables; Table S4: Three-way PERMANOVA analysis and pair-wise tests for development and bioconversion variables.
