# Supplementary material for: The Influence of Non-Optimal Rearing Conditions and Substrates on the Performance of the Black Soldier Fly (Hermetia illucens)
Source: Insects. 2022 Jul 16;13(7):639. doi: 10.3390/insects13070639 (PMC9320413; doi:10.3390/insects13070639)
Supplement: Supplementary file 1 [file insects-13-00639-s001.zip › insects-1802561-supplementary.pdf]

Supplementary Online Material of MDPI – Insects

Influence of non-optimal rearing conditions and substrates on the performance of Black Soldier Fly (Hermetia illucens)

Nuno Ribeiro. Rui Costa & Olga M. C. C. Ameixa

Table S1 - Black soldier fly development parameters (mean ± SD); WRI – substrate moisture; GR – growth rate

Substrate	Temp (°C)	SM (%H_2O)	Feed[1] (mg/larva.d)	Survival[2] (%)	Total Biomass[2] (g/larva)	GR[2] (mg/d)
**CONTROL**			5.696±0.022	33.0±10.3a	0.167±0.021a	1.513±0.193a
**Pumpkin**			15.807±0.111	97.6±2.9b	0.113±0.008b	1.814±0.229b
**Apple**			16.068±0.561	87.0±8.3c	0.036±0.004c	0.259±0.027c
**Pomace**	20		9.451±0.508	47.6±7.5d	0.004±0.000d	0.029±0.003d
**Red Onion**			17.739±0.185	80.0±13.3c	0.092±0.010e	1.282±0.142e
**Red Cabbage**			23.632±0.221	79.0±6.5c	0.095±0.008f	1.183±0.106e
**Spinach**			13.410±0.013	12.0±2.3e	0.070±0.100e	0.690±0.158f
**CONTROL**			6.736±0.030	91.4±6.0ac	0.152±0.006a	1.589±0.067a
**Pumpkin**			17.893±0.279	97.6±1.5b	0.121±0.003b	2.621±0.055b
**Apple**			14.232±0.366	87.2±5.0a	0.076±0.004c	0.564±0.032c
**Pomace**	25	70	9.967±0.554	87.2±7.1a	0.025±0.003d	0.212±0.024d
**Red Onion**			7.312±0.856	93.4±7.4ab	0.131±0.006b	2.103±0.081ab
**Red Cabbage**			17.676±0.014	96.4±1.1bc	0.110±0.004b	2.241±0.138b
**Spinach**			7.470±0.016	20.2±4.7d	0.057±0.006c	0.509±0.055e
**CONTROL**			5.134±0.526	86.8±2.7a	0.133±0.007a	1.655±0.087a
**Pumpkin**			8.793±0.011	98.2±2.7b	0.111±0.005b	2.616±0.145be
**Apple**			12.545±0.038	91.8±3.7ad	0.067±0.003c	0.495±0.023c
**Pomace**	30		7.781±0.049	57.6±9.2c	0.008±0.001d	0.058±0.001d
**Red Onion**			12.846±0.855	87.6±10.4ab	0.123±0.009ae	2.217±0.177b
**Red Cabbage**			20.785±0.012	96.8±3.3bd	0.121±0.011e	1.828±0.174e
**Spinach**			12.045±0.055	2.8±2.2e	0.049±0.009f	0.551±0.110f
**CONTROL**			5.125±0.012	33.0±20.0a	0.148±0.028a	1.328±0.026ac
**Pumpkin**			14.631±0.018	82.6±10.4bde	0.103±0.008b	1.577±0.124a
**Apple**			19.987±0.027	81.6±4.7b	0.036±0.002c	0.260±0.012b
**Pomace**	20		13.183±0.027	0c	0	0
**Red Onion**			15.990±0.012	68.4±8.1d	0.070±0.007d	1.122±0.112cd
**Red Cabbage**			21.134±0.025	94.0±4.5e	0.086±0.004e	1.041±0.046d
**Spinach**			14.108±0.011	37.2±2.9a	0.029±0.002f	0.256±0.018b
**CONTROL**			6.614±0.473	46.2±10.2a	0.158±0.007a	1.438±0.062a
**Pumpkin**			16.121±0.031	95.8±1.3b	0.109±0.03b	1.686±0.042b
**Apple**			21.689±0.030	92.4±4.3b	0.051±0.006c	0.374±0.045c
**Pomace**	25	NAT	13.341±0.026	19.0±6.6c	0.011±0.001	0.090±0.010
**Red Onion**			5.696±0.022	91.2±5.3b	0.089±0.005d	1.432±0.074a
**Red Cabbage**			15.807±0.111	93.6±2.3b	0.137±0.003e	1.657±0.043b
**Spinach**			16.068±0.561	51.6±16.8a	0.100±0.022bd	0.917±0.201d
**CONTROL**			9.451±0.508	53.2±11.6a	0.144±0.017ade	1.317±0.156a
**Pumpkin**			17.739±0.185	98.0±1.4b	0.092±0.005b	1.450±0.085b
**Apple**			23.632±0.221	89.4±4.8c	0.052±0.002c	0.382±0.018c
**Pomace**	30		13.410±0.013	0d	0	0
**Red Onion**			6.736±0.030	76.2±13.5e	0.113±+0.022d	1.812±0.346d
**Red Cabbage**			17.893±0.279	96.4±3.0b	0.139±0.003e	1.684±0.033d
**Spinach**			14.232±0.366	16.6±3.0e	0.052±0.012e	0.484±0.112e

[1] Dry matter basis [2] Means in a column. within groups of TempxSM. which are followed by the same letter are not significantly different (P≤0.05)

Table S2 - 3-way Permanova analysis and pair-wise tests for survival results (all substrates)

PERMANOVA SURVIVAL

Factors

**Name**	**Abbrev.**	**Type**	**Levels**
Subs	Su	Fixed	7
Temp	Te	Fixed	3
RH	RH	Fixed	2

PERMANOVA table of results

Source	df	SS	MS	Pseudo-F	P(perm)	Unique perms
Su	6	15.43	2.5717	457	0.0001	9945
Te	2	0.94655	0.47327	84	0.0001	9943
RH	1	0.54927	0.54927	98	0.0001	9833
SuxTe	12	1.0582	0.088182	16	0.0001	9932
SuxRH	6	2.976	0.496	88	0.0001	9943
TexRH	2	0.05674	0.028373	5	0.0058	9953
SuxTexRH	12	0.42069	0.035057	6.2	0.0001	9931
Res	168	0.94616	0.0056319			
Total	209	22.384				

PAIR-WISE TESTS **Term 'SuxTexRH' for pairs of levels of factor 'Subs'**

Within level '20' of factor 'Temp'
Within level '70' of factor 'RH'

Groups	t	P(perm)	Unique perms
CONTROL, Pumpkin	13.511	0.0086	51
CONTROL, Apple	9.1147	0.0083	78
CONTROL, Pomace	2.5626	0.0486	40
CONTROL, Red Onion	6.2417	0.008	70
CONTROL, Red Cabbage	8.4407	0.0074	70
CONTROL, Spinach	4.447	0.0071	42
Pumpkin, Apple	2.6872	0.0345	28
Pumpkin, Pomace	13.91	0.0078	54
Pumpkin, Red Onion	2.8872	0.0076	37
Pumpkin, Red Cabbage	5.8353	0.0084	36
Pumpkin. Spinach	51.525	0.0071	29
Apple, Pomace	7.8549	0.0079	57
Apple, Red Onion	0.99594	0.3759	35
Apple, Red Cabbage	1.6903	0.1296	29
Apple, Spinach	19.365	0.008	51
Pomace, Red Onion	4.7381	0.0068	55
Pomace, Red Cabbage	7.0638	0.0068	55
Pomace, Spinach	10.126	0.0084	50
Red Onion, Red Cabbage	0.15076	0.8447	35
Red Onion, Spinach	11.24	0.0083	60
Red Cabbage, Spinach	21.624	0.0083	45

Within level '20' of factor 'Temp'
Within level 'N' of factor 'RH'

Groups	t	P(perm)	Unique perms
CONTROL, Pumpkin	4.9242	0.0072	72
CONTROL, Apple	5.2977	0.0075	75
CONTROL, Pomace	3.6964	0.0077	14
CONTROL, Red Onion	3.6736	0.0085	53
CONTROL. Red Cabbage	6.6636	0.0073	70
CONTROL, Spinach	0.4654	0.6813	39
Pumpkin, Apple	0.19529	0.8446	27
Pumpkin, Pomace	17.707	0.0088	16
Pumpkin, Red Onion	2.403	0.0662	32
Pumpkin, Red Cabbage	2.2418	0.0801	35
Pumpkin. Spinach	9.3653	0.0072	52
Apple, Pomace	38.639	0.008	15
Apple, Red Onion	3.1446	0.0256	24
Apple, Red Cabbage	4.2382	0.0153	29
Apple, Spinach	17.831	0.0083	38
Pomace, Red Onion	18.855	0.0081	9
Pomace, Red Cabbage	46.423	0.0098	12
Pomace, Spinach	28.201	0.0091	11
Red Onion, Red Cabbage	6.162	0.0084	26
Red Onion, Spinach	8.0828	0.0091	35
Red Cabbage, Spinach	23.504	0.0069	40

Within level '25' of factor 'Temp'
Within level '70' of factor 'RH'

Groups	t	P(perm)	Unique perms
CONTROL, Pumpkin	2.2314	0.0301	17
CONTROL, Apple	1.1649	0.2934	23
CONTROL, Pomace	1.0098	0.3561	25
CONTROL, Red Onion	0.46855	0.6445	24
CONTROL, Red Cabbage	1.8233	0.0935	15
CONTROL, Spinach	20.905	0.0086	57
Pumpkin, Apple	4.1767	0.0086	24
Pumpkin, Pomace	3.2095	0.0472	23
Pumpkin, Red Onion	1.2428	0.3221	13
Pumpkin, Red Cabbage	1.4142	0.2829	7
Pumpkin. Spinach	35.328	0.0102	36
Apple, Pomace	5.24E-09	1	22
Apple, Red Onion	1.5172	0.1711	17
Apple, Red Cabbage	3.7559	0.0072	23
Apple, Spinach	21.103	0.0068	53
Pomace, Red Onion	1.353	0.2411	27
Pomace, Red Cabbage	2.8666	0.0441	25

Within level '25' of factor 'Temp'
Within level 'N' of factor 'RH'

Groups	t	P(perm)	Unique perms
CONTROL, Pumpkin	10.829	0.0081	45
CONTROL, Apple	9.3721	0.0061	48
CONTROL, Pomace	5.013	0.0145	43
CONTROL, Red Onion	8.7948	0.0088	54
CONTROL, Red Cabbage	10.175	0.0095	44
CONTROL, Spinach	0.61419	0.5948	42
Pumpkin, Apple	1.7	0.0856	11
Pumpkin, Pomace	25.403	0.009	42
Pumpkin, Red Onion	1.897	0.1313	16
Pumpkin, Red Cabbage	1.8593	0.1539	9
Pumpkin. Spinach	5.8544	0.0066	63
Apple, Pomace	20.794	0.009	46
Apple, Red Onion	0.39563	0.7305	19
Apple, Red Cabbage	0.55234	0.736	11
Apple, Spinach	5.2533	0.0086	58
Pomace, Red Onion	19.066	0.0069	49
Pomace, Red Cabbage	23.757	0.0072	35

Pomace, Spinach	17.668	0.0077	58
Red Onion, Red Cabbage	0.89562	0.55	14
Red Onion, Spinach	18.714	0.0079	44
Red Cabbage, Spinach	35.528	0.0102	39

Within level '30' of factor 'Temp'
Within level '70' of factor 'RH'

**Groups**	**t**	**P(perm)**	**Unique perms**
CONTROL, Pumpkin	6.7175	0.0076	21
CONTROL, Apple	2.4456	0.0578	15
CONTROL, Pomace	6.7889	0.008	49
CONTROL, Red Onion	0.16609	0.9307	23
CONTROL, Red Cabbage	5.2852	0.0077	23
CONTROL, Spinach	54.449	0.0087	30
Pumpkin, Apple	3.1303	0.0245	18
Pumpkin, Pomace	9.4393	0.0075	47
Pumpkin, Red Onion	2.2007	0.0611	30
Pumpkin, Red Cabbage	0.73992	0.6041	7
Pumpkin. Spinach	61.839	0.0086	27
Apple, Pomace	7.6859	0.009	61
Apple, Red Onion	0.84853	0.4987	26
Apple, Red Cabbage	2.2634	0.0753	16
Apple, Spinach	46.394	0.0082	36
Pomace, Red Onion	4.815	0.0085	54
Pomace, Red Cabbage	8.9461	0.0076	58
Pomace, Spinach	12.916	0.0067	56
Red Onion, Red Cabbage	1.8819	0.1059	31
Red Onion, Spinach	17.798	0.0095	59
Red Cabbage, Spinach	53.561	0.008	33

PAIR-WISE TESTS
Term 'SuxTexRH' for pairs of levels of factor 'Temp'

Within level 'CONTROL' of factor 'Subs'
Within level '70' of factor 'RH'

Groups	t	P(perm)	Unique perms
20, 25	10.947	0.0072	72
20, 30	11.307	0.0099	57
25, 30	1.5595	0.1683	19

Within level 'Pumpkin' of factor 'Subs'
Within level '70' of factor 'RH'

Groups	t	P(perm)	Unique perms
20, 25	Negative		
20, 30	0.34078	0.8479	10
25, 30	0.43529	0.7683	9

Within level 'Apple' of factor 'Subs'
Within level '70' of factor 'RH'

Groups	t	P(perm)	Unique perms
20, 25	0.045129	1	25
20, 30	1.1767	0.3062	24
25, 30	1.5796	0.1878	18

Within level 'Pomace' of factor 'Subs'
Within level '70' of factor 'RH'

Groups	t	P(perm)	Unique perms
20, 25	8.5804	0.0081	62
20, 30	1.8791	0.1101	33
25, 30	5.686	0.0073	49

Within level 'Red Onion' of factor 'Subs'
Within level '70' of factor 'RH'

Groups	t	P(perm)	Unique perms
20, 25	1.9659	0.0831	24
20, 30	1.0044	0.3663	39
25, 30	1.014	0.3844	31

Pomace, Spinach	4.0293	0.0096	57
Red Onion, Red Cabbage	0.9342	0.4251	16
Red Onion, Spinach	5.0211	0.0095	66
Red Cabbage, Spinach	5.5282	0.0087	58

Within level '30' of factor 'Temp'
Within level 'N' of factor 'RH'

**Groups**	**t**	**P(perm)**	**Unique perms**
CONTROL, Pumpkin	8.568	0.007	41
CONTROL, Apple	6.4397	0.0081	54
CONTROL, Pomace	10.25	0.0095	15
CONTROL, Red Onion	2.8913	0.0258	50
CONTROL, Red Cabbage	8.0498	0.0066	65
CONTROL, Spinach	6.8558	0.0094	56
Pumpkin, Apple	3.8232	0.0096	20
Pumpkin, Pomace	154.95	0.0074	7
Pumpkin, Red Onion	3.5966	0.0085	25
Pumpkin, Red Cabbage	1.0643	0.3844	9
Pumpkin. Spinach	58.143	0.0072	23
Apple, Pomace	41.414	0.0084	15
Apple, Red Onion	2.0615	0.0776	39
Apple, Red Cabbage	2.7414	0.0222	19
Apple, Spinach	29.19	0.0074	41
Pomace, Red Onion	12.64	0.0082	16
Pomace, Red Cabbage	70.684	0.0085	14
Pomace, Spinach	13.291	0.0054	12
Red Onion, Red Cabbage	3.2683	0.0228	35
Red Onion, Spinach	9.6811	0.0085	59
Red Cabbage, Spinach	43.151	0.0084	35

Within level 'CONTROL' of factor 'Subs'
Within level 'N' of factor 'RH'

Groups	t	P(perm)	Unique perms
20, 25	1.3178	0.2532	50
20, 30	1.9561	0.0927	58
25, 30	1.0148	0.3756	33

Within level 'Pumpkin' of factor 'Subs'
Within level 'N' of factor 'RH'

Groups	t	P(perm)	Unique perms
20, 25	2.8079	0.0217	27
20, 30	3.2714	0.0084	30
25, 30	2.5574	0.0508	7

Within level 'Apple' of factor 'Subs'
Within level 'N' of factor 'RH'

Groups	t	P(perm)	Unique perms
20, 25	3.7901	0.0255	25
20, 30	2.5828	0.0478	24
25, 30	1.0401	0.3988	16

Within level 'Pomace' of factor 'Subs'
Within level 'N' of factor 'RH'

Groups	t	P(perm)	Unique perms
20, 25	6.4049	0.0092	12
20, 30		Denominator is 0	
25, 30	6.4049	0.0088	12

Within level 'Red Onion' of factor 'Subs'
Within level 'N' of factor 'RH'

Groups	t	P(perm)	Unique perms
20, 25	5.2725	0.0065	31
20, 30	1.1086	0.3417	34
25, 30	2.3179	0.0426	38

Within level 'Red Cabbage' of factor 'Subs'
Within level '70' of factor 'RH'

Groups	t	P(perm)	Unique perms
20, 25	5.8789	0.0079	31
20, 30	5.4569	0.0072	34
25, 30	0.2582	0.9038	10

Within level 'Red Cabbage' of factor 'Subs'
Within level 'N' of factor 'RH'

Groups	t	P(perm)	Unique perms
20, 25	0.17609	0.9296	13
20, 30	0.98308	0.4237	15
25, 30	1.6386	0.1507	12

Within level 'Spinach' of factor 'Subs'
Within level '70' of factor 'RH'

Groups	t	P(perm)	Unique perms
20, 25	3.5157	0.0235	19
20, 30	6.4413	0.0089	22
25, 30	7.5724	0.0079	35

Within level 'Spinach' of factor 'Subs'
Within level 'N' of factor 'RH'

Groups	t	P(perm)	Unique perms
20, 25	1.8843	0.0782	39
20, 30	11.34	0.0092	33
25, 30	4.587	0.0089	56

Pairwise test Term 'SuxTexRH' for pairs of levels of factor 'RH'

Within level 'CONTROL' of factor 'Subs'
Within level '20' of factor 'Temp'

Groups	t	P(perm)	Unique perms
70. N	Negative		

Within level 'CONTROL' of factor 'Subs'
Within level '25' of factor 'Temp'

Groups	t	P(perm)	Unique perms
70. N	8.5573	0.0108	57

Within level 'CONTROL' of factor 'Subs'
Within level '30' of factor 'Temp'

Groups	t	P(perm)	Unique perms
70. N	6.3071	0.0083	52

Within level 'Pumpkin' of factor 'Subs'
Within level '20' of factor 'Temp'

Groups	t	P(perm)	Unique perms
70. N	3.0995	0.0247	34

Within level 'Pumpkin' of factor 'Subs'
Within level '25' of factor 'Temp'

Groups	t	P(perm)	Unique perms
70. N	2.0125	0.1198	7

Within level 'Pumpkin' of factor 'Subs'
Within level '30' of factor 'Temp'

Groups	t	P(perm)	Unique perms
70. N	0.14744	1	8

Within level 'Apple' of factor 'Subs'
Within level '20' of factor 'Temp'

Groups	t	P(perm)	Unique perms
70. N	1.2603	0.2416	25

Within level 'Apple' of factor 'Subs'
Within level '25' of factor 'Temp'

Groups	t	P(perm)	Unique perms
70. N	1.6961	0.1174	19

Within level 'Apple' of factor 'Subs'
Within level '30' of factor 'Temp'

Groups	t	P(perm)	Unique perms
70. N	0.88226	0.4827	15

Within level 'Pomace' of factor 'Subs'
Within level '20' of factor 'Temp'

Groups	t	P(perm)	Unique perms
70. N	14.185	0.0075	15

Within level 'Pomace' of factor 'Subs'
Within level '25' of factor 'Temp'

Groups	t	P(perm)	Unique perms
70. N	15.712	0.009	58

Within level 'Pomace' of factor 'Subs'
Within level '30' of factor 'Temp'

Groups	t	P(perm)	Unique perms
70. N	13.945	0.0092	15

Within level 'Red Onion' of factor 'Subs'
Within level '20' of factor 'Temp'

Groups	t	P(perm)	Unique perms
70. N	1.6629	0.1939	27

Within level 'Red Onion' of factor 'Subs'
Within level '25' of factor 'Temp'

Groups	t	P(perm)	Unique perms
70. N	0.5416	0.6211	21

Within level 'Red Onion' of factor 'Subs'
Within level '30' of factor 'Temp'

Groups	t	P(perm)	Unique perms
70. N	1.4956	0.1852	43

Within level 'Red Cabbage' of factor 'Subs'
Within level '20' of factor 'Temp'

Groups	t	P(perm)	Unique perms
70. N	4.2258	0.017	33

Within level 'Red Cabbage' of factor 'Subs'
Within level '25' of factor 'Temp'

Within level 'Red Cabbage' of factor 'Subs'
Within level '30' of factor 'Temp'

Groups	t	P(perm)	Unique perms
70. N	2.4371	0.0692	10

Groups	t	P(perm)	Unique perms
70. N	0.2	0.9258	13

Within level 'Spinach' of factor 'Subs'
Within level '20' of factor 'Temp'

Groups	t	P(perm)	Unique perms
70. N	14.953	0.0083	32

Within level 'Spinach' of factor 'Subs'
Within level '25' of factor 'Temp'

Groups	t	P(perm)	Unique perms
70. N	4.0204	0.0085	59

Within level 'Spinach' of factor 'Subs'
Within level '30' of factor 'Temp'

Groups	t	P(perm)	Unique perms
70. N	8.7279	0.0083	26

Table S3 - 2-way Permanova analysis and pair-wise tests for development and bioconversion variables (all substrates, 70 %SM). Variables – Weight; Waste Reduction Index (WRI); Feed Conversion Ratio (FCR); Bioconversion Ratio (BR); (Efficiency of conversion of digested feed (ECD); Growth Rate (GR)

PERMANOVA WEIGHT

Factors

Name	Abbrev.	Type	Levels
Subs	Su	Fixed	7
Temp	Te	Fixed	3

PERMANOVA table of results

Source	df	SS	MS	Pseudo-F	P(perm)	Unique perms
Su	6	0.40359	0.067265	338.29	0.0001	9937
Te	2	0.014015	0.0070077	35.244	0.0001	9943
SuxTe	12	0.017756	0.0014796	7.4414	0.0001	9929
Res	189	0.03758	0.0001988			
Total	209	0.47294				

PAIR-WISE TESTS **Term 'SuxTe' for pairs of levels of factor 'Subs'**

Within level '20' of factor 'Temp'

Groups	t	P(perm)	Unique perms
CONTROL, Pumpkin	5.8142	0.0002	9350
CONTROL, Apple	15.382	0.0001	9297
CONTROL, Pomace	19.716	0.0002	6466
CONTROL, Red Onion	8.4519	0.0001	9329
CONTROL, Red Cabbage	8.0613	0.0001	9311
CONTROL, Spinach	10.13	0.0001	9344
Pumpkin, Apple	23.824	0.0001	9162
Pumpkin, Pomace	35.338	0.0001	6326
Pumpkin, Red Onion	5.1691	0.0003	9297
Pumpkin, Red Cabbage	4.5891	0.0003	9339
Pumpkin. Spinach	7.6117	0.0001	9335
Apple, Pomace	30.583	0.0001	6346
Apple, Red Onion	10.075	0.0001	9295
Apple, Red Cabbage	20.76	0.0001	9233
Apple, Spinach	1.8392	0.075	9294
Pomace, Red Onion	17.716	0.0001	6554
Pomace, Red Cabbage	34.075	0.0001	6430
Pomace, Spinach	6.5209	0.0001	6609
Red Onion, Red Cabbage	1.9074	0.0725	9360
Red Onion, Spinach	3.7719	0.0024	9307
Red Cabbage, Spinach	5.4433	0.0002	9304

Within level '25' of factor 'Temp'

Groups	t	P(perm)	Unique perms
CONTROL, Pumpkin	11.963	0.0002	9359
CONTROL, Apple	17.805	0.0001	9234
CONTROL, Pomace	40.375	0.0001	8811
CONTROL, Red Onion	5.7695	0.0001	9301
CONTROL, Red Cabbage	6.1001	0.0001	9314
CONTROL, Spinach	8.5268	0.0001	9329
Pumpkin, Apple	10.2	0.0001	9315
Pumpkin, Pomace	30.002	0.0001	8994
Pumpkin, Red Onion	0.56972	0.5583	9332
Pumpkin, Red Cabbage	1.6042	0.1246	9329
Pumpkin. Spinach	4.1026	0.0014	9328
Apple, Pomace	9.115	0.0001	9354
Apple, Red Onion	5.4797	0.0001	9336
Apple, Red Cabbage	9.2538	0.0001	9306
Apple, Spinach	1.4943	0.1585	9308
Pomace, Red Onion	12.159	0.0001	9309
Pomace, Red Cabbage	20.483	0.0001	9219
Pomace, Spinach	6.7733	0.0001	9323
Red Onion, Red Cabbage	1.4576	0.1667	9334
Red Onion, Spinach	2.8586	0.012	9341
Red Cabbage, Spinach	4.5823	0.0004	9321

Within level '30' of factor 'Temp'

Groups	t	P(perm)	Unique perms
CONTROL, Pumpkin	6.8213	0.0001	9327
CONTROL, Apple	17.516	0.0001	9224
CONTROL, Pomace	36.013	0.0001	6284
CONTROL, Red Onion	2.6677	0.0157	9339
CONTROL, Red Cabbage	1.0497	0.3063	9318
CONTROL, Spinach	18.134	0.0001	9280
Pumpkin, Apple	9.5572	0.0002	9283
Pumpkin, Pomace	26.169	0.0001	6405
Pumpkin, Red Onion	2.635	0.0151	9376
Pumpkin, Red Cabbage	5.465	0.0002	9337
Pumpkin. Spinach	10.777	0.0001	9337
Apple, Pomace	19.316	0.0001	6501
Apple, Red Onion	9.9554	0.0002	9340
Apple, Red Cabbage	15.301	0.0001	9277
Apple, Spinach	2.2231	0.0381	9318
Pomace, Red Onion	21.132	0.0001	6533
Pomace, Red Cabbage	31.772	0.0001	6392
Pomace, Spinach	13.778	0.0001	6559
Red Onion, Red Cabbage	1.7655	0.0867	9321
Red Onion, Spinach	11.008	0.0001	9305
Red Cabbage, Spinach	16.11	0.0001	9266

PAIR-WISE TESTS **Term 'SuxTe' for pairs of levels of factor 'Temp'**

Within level 'CONTROL' of factor 'Subs'

Groups	t	P(perm)	Unique perms
20, 25	0.21106	0.8318	9328
20, 30	2.5317	0.0256	9350
25, 30	4.7049	0.0002	9304

Within level 'Pumpkin' of factor 'Subs'

Groups	t	P(perm)	Unique perms
20, 25	1.8299	0.0801	9289
20, 30	1.441	0.1703	9310
25, 30	3.191	0.0058	9310

Within level 'Apple' of factor 'Subs'

Groups	t	P(perm)	Unique perms
20, 25	6.1197	0.0001	930/8
20, 30	8.7164	0.0001	9310
25, 30	0.77568	0.4425	9361

Within level 'Pomace' of factor 'Subs'

Groups	t	P(perm)	Unique perms
20, 25	6.4116	0.0001	6664
20, 30	1.1122	0.2927	505
25, 30	5.2757	0.0001	6566

Within level 'Red Onion' of factor 'Subs'

Groups	t	P(perm)	Unique perms
20, 25	3.4905	0.0035	9361
20, 30	5.4461	0.0001	9345
25, 30	0.85855	0.3963	9333

Within level 'Red Cabbage' of factor 'Subs'

Groups	t	P(perm)	Unique perms
20, 25	6.2331	0.0001	9310
20, 30	8.6813	0.0002	9353
25, 30	1.1143	0.2853	9350

Within level 'Spinach' of factor 'Subs'

Groups	t	P(perm)	Unique perms
20, 25	2.5944	0.01982	9358
20, 30	0.18795	0.8503	9248
25, 30	3.0088	0.0043	9299

PERMANOVA Waste Reduction Index (WRI)

Factors

**Name**	**Abbrev.**	**Type**	**Levels**
Subs	Su	Fixed	7
Temp	Te	Fixed	3

PERMANOVA table of results

Source	df	SS	MS	Pseudo-F	P(perm)	Unique perms
Su	6	31.27	5.2117	336.84	0.0001	9949
Te	2	1.1611	0.58055	37.522	0.0001	9947
SuxTe	12	0.92334	0.076945	4.9732	0.0001	9929
Res	189	2.9242	0.015472			
Total	209	36.279				

PAIR-WISE TESTS **Term 'SuxTe' for pairs of levels of factor 'Subs'**

Within level '20' of factor 'Temp'

Groups	t	P(perm)	Unique perms
CONTROL, Pumpkin	23.088	0.0001	9166
CONTROL, Apple	0.65851	0.5106	9367
CONTROL, Pomace	4.8679	0.0003	9322
CONTROL, Red Onion	16.506	0.0001	9295
CONTROL, Red Cabbage	10.851	0.0001	9319
CONTROL, Spinach	1.5243	0.1368	9333
Pumpkin, Apple	22.151	0.0001	9137
Pumpkin, Pomace	24.945	0.0001	9117
Pumpkin, Red Onion	0.7762	0.4413	9331
Pumpkin, Red Cabbage	9.2617	0.0001	9378
Pumpkin. Spinach	14.111	0.0001	9260
Apple, Pomace	3.4825	0.0042	9305
Apple, Red Onion	16.317	0.0001	9282
Apple, Red Cabbage	10.603	0.0002	9361
Apple, Spinach	1.7734	0.0947	9352
Pomace, Red Onion	18.032	0.0001	9305
Pomace, Red Cabbage	13.092	0.0001	9320
Pomace, Spinach	3.614	0.0012	9298
Red Onion, Red Cabbage	7.9315	0.0001	9313
Red Onion, Spinach	12.142	0.0001	9340

Within level '25' of factor 'Temp'

Groups	t	P(perm)	Unique perms
CONTROL, Pumpkin	16.206	0.0001	9239
CONTROL, Apple	3.5802	0.0021	9381
CONTROL, Pomace	6.4056	0.0001	9329
CONTROL, Red Onion	19.884	0.0001	9274
CONTROL, Red Cabbage	7.0988	0.0001	9353
CONTROL, Spinach	0.56795	0.5754	9292
Pumpkin, Apple	21.474	0.0001	9293
Pumpkin, Pomace	22.508	0.0001	9206
Pumpkin, Red Onion	0.078825	0.9487	9346
Pumpkin, Red Cabbage	0.68821	0.4969	9290
Pumpkin. Spinach	17.869	0.0002	9285
Apple, Pomace	5.7672	0.0003	9269
Apple, Red Onion	29.268	0.0001	8976
Apple, Red Cabbage	8.348	0.0001	9368
Apple, Spinach	7.3305	0.0001	9373
Pomace, Red Onion	29.267	0.0001	9077
Pomace, Red Cabbage	9.2842	0.0001	9359
Pomace, Spinach	10.003	0.0001	9243
Red Onion, Red Cabbage	0.75271	0.4586	9308
Red Onion, Spinach	23.541	0.0002	9159

Red Cabbage, Spinach	5.9549	0.0002	9306	Red Cabbage, Spinach	7.1071	0.0001	9373

Within level '30' of factor 'Temp'

Groups	t	P(perm)	Unique perms
CONTROL, Pumpkin	7.1614	0.0001	9286
CONTROL, Apple	3.5814	0.0001	9402
CONTROL, Pomace	4.3317	0.0002	9326
CONTROL, Red Onion	12.075	0.0001	9387
CONTROL, Red Cabbage	6.4667	0.0002	9332
CONTROL, Spinach	1.8194	0.0819	9333
Pumpkin, Apple	11.481	0.0001	9388
Pumpkin, Pomace	11.868	0.0001	9353
Pumpkin, Red Onion	0.12639	0.8896	9300
Pumpkin, Red Cabbage	3.9809	0.0001	9426
Pumpkin. Spinach	9.1742	0.0001	9287
Apple, Pomace	2.6809	0.0153	9308
Apple, Red Onion	71.55	0.0001	8317
Apple, Red Cabbage	41.579	0.0001	8791
Apple, Spinach	1.642	0.1162	9306
Pomace, Red Onion	43.15	0.0003	8790
Pomace, Red Cabbage	27.654	0.0001	9073
Pomace, Spinach	2.6608	0.0191	9320
Red Onion, Red Cabbage	19.855	0.0001	9184
Red Onion, Spinach	18.163	0.0002	9250
Red Cabbage, Spinach	11.011	0.0001	9355

PAIR-WISE TESTS **Term 'SuxTe' for pairs of levels of factor 'Temp'**

Within level 'CONTROL' of factor 'Subs'

Groups	t	P(perm)	Unique perms
20, 25	4.3512	0.0002	9381
20, 30	4.2823	0.0001	9398
25, 30	2.0676	0.055	9392

Within level 'Pumpkin' of factor 'Subs'

Groups	t	P(perm)	Unique perms
20, 25	3.0381	0.009	932
20, 30	2.6109	0.016	9364
25, 30	0.79665	0.4312	9359

Within level 'Apple' of factor 'Subs'

Groups	t	P(perm)	Unique perms
20, 25	2.3157	0.0275	9317
20, 30	3.2882	0.0045	9320
25, 30	1.8017	0.0911	9336

Within level 'Pomace' of factor 'Subs'

Groups	t	P(perm)	Unique perms
20, 25	0.58178	0.5658	9341
20, 30	2.9668	0.0106	9300
25, 30	2.3279	0.0376	9353

Within level 'Red Onion' of factor 'Subs'

Groups	t	P(perm)	Unique perms
20, 25	2.0672	0.0547	9343
20, 30	4.003	0.0001	9396
25, 30	2.4942	0.0247	9384

Within level 'Red Cabbage' of factor 'Subs'

Groups	t	P(perm)	Unique perms
20, 25	4.7679	0.0001	9363
20, 30	9.0714	0.0001	9361
25, 30	1.8753	0.0641	9375

Within level 'Spinach' of factor 'Subs'

Groups	t	P(perm)	Unique perms
20, 25	1.9793	0.0621	9302
20, 30	1.013	0.3112	9298
25, 30	0.40755	0.6839	9351

PERMANOVA Feed Conversion Ratio (FCR)

Factors

**Name**	**Abbrev.**	**Type**	**Levels**
Subs	Su	Fixed	7
Temp	Te	Fixed	3

PERMANOVA table of results

Source	df	SS	MS	Pseudo-F	P(perm)	Unique perms
Su	6	203.94	33.989	207.37	0.0001	9940
Te	2	9.7723	4.8861	29.81	0.0001	9967
SuxTe	12	21.72	1.81	11.043	0.0001	9952
Res	189	30.979	0.16391			
Total	209	266.41				

PAIR-WISE TESTS **Term 'SuxTe' for pairs of levels of factor 'Subs'**

Within level '20' of factor 'Temp'

Groups	t	P(perm)	Unique perms
CONTROL, Pumpkin	4.7717	0.0001	9400
CONTROL, Apple	4.5095	0.0005	9405
CONTROL, Pomace	0.12554	0.0001	6676
CONTROL, Red Onion	4.2948	0.0001	9392
CONTROL, Red Cabbage	2.9578	0.0011	9372
CONTROL, Spinach	11.078	0.0001	9376
Pumpkin, Apple	32.683	0.0001	9014
Pumpkin, Pomace	0.92104	0.0001	6629
Pumpkin, Red Onion	1.1562	0.2451	9330
Pumpkin, Red Cabbage	3.8058	0.0028	9317
Pumpkin. Spinach	50.825	0.0001	8646
Apple, Pomace	0.62016	0.0001	6637
Apple, Red Onion	26.205	0.0003	9159
Apple, Red Cabbage	17.747	0.0001	9282
Apple, Spinach	22.169	0.0001	9199
Pomace, Red Onion	0.85331	0.0001	6630
Pomace, Red Cabbage	0.64304	0.0001	6653
Pomace, Spinach	1.7243	0.0001	6627
Red Onion, Red Cabbage	2.6553	0.0171	9367
Red Onion, Spinach	42.68	0.0001	8785
Red Cabbage, Spinach	31.766	0.0001	8992

Within level '25' of factor 'Temp'

Groups	t	P(perm)	Unique perms
CONTROL, Pumpkin	5.4184	0.0001	9308
CONTROL, Apple	7.3488	0.0001	9278
CONTROL, Pomace	5.9107	0.0001	9338
CONTROL, Red Onion	4.3766	0.0001	9380
CONTROL, Red Cabbage	1.071	0.3098	9393
CONTROL, Spinach	7.6072	0.0001	9322
Pumpkin, Apple	42.206	0.0001	8730
Pumpkin, Pomace	7.6596	0.0001	9357
Pumpkin, Red Onion	3.3711	0.0053	9345
Pumpkin, Red Cabbage	4.2095	0.0001	9327
Pumpkin. Spinach	11.909	0.0001	9391
Apple, Pomace	3.9749	0.0001	9394
Apple, Red Onion	30.78	0.0001	8979
Apple, Red Cabbage	9.2743	0.0001	9306
Apple, Spinach	4.2912	0.0001	9371
Pomace, Red Onion	7.3857	0.0001	9392
Pomace, Red Cabbage	6.3344	0.0001	9335
Pomace, Spinach	1.687	0.1042	9311
Red Onion, Red Cabbage	3.1334	0.0105	9414
Red Onion, Spinach	11.268	0.0001	9375
Red Cabbage, Spinach	8.4679	0.0002	9325

Within level '30' of factor 'Temp'

Groups	t	P(perm)	Unique perms
CONTROL, Pumpkin	3.3853	0.003	9332
CONTROL, Apple	5.8789	0.0001	9325
CONTROL, Pomace	1.2001	0.0001	6692
CONTROL, Red Onion	4.4894	0.0001	9362
CONTROL, Red Cabbage	2.9031	0.0128	9308
CONTROL, Spinach	8.8085	0.0001	9380
Pumpkin, Apple	10.125	0.0002	9294
Pumpkin, Pomace	0.46452	0.0001	6609
Pumpkin, Red Onion	0.081171	0.9306	9367
Pumpkin, Red Cabbage	0.52502	0.6309	9153
Pumpkin. Spinach	10.151	0.0001	9326
Apple, Pomace	2.2308	0.0003	6660
Apple, Red Onion	17.265	0.0002	9240
Apple, Red Cabbage	9.6679	0.0001	9327
Apple, Spinach	7.1716	0.0001	9334
Pomace, Red Onion	0.45536	0.0001	6697
Pomace, Red Cabbage	0.57769	0.0001	6580
Pomace, Spinach	5.377	0.0108	6660
Red Onion, Red Cabbage	0.77189	0.4398	9314
Red Onion, Spinach	10.522	0.0001	9331
Red Cabbage, Spinach	9.9645	0.0003	9386

PAIR-WISE TESTS **Term 'SuxTe' for pairs of levels of factor 'Temp'**

Within level 'CONTROL' of factor 'Subs'

Groups	t	P(perm)	Unique perms
20, 25	2.579	0.0093	9312
20, 30	0.44157	0.6814	9325
25, 30	3.8524	0.0012	9326

Within level 'Pumpkin' of factor 'Subs'

Groups	t	P(perm)	Unique perms
20, 25	8.0937	0.0002	9307
20, 30	3.1189	0.0089	9339
25, 30	5.9276	0.0001	9348

Within level 'Apple' of factor 'Subs'

Groups	t	P(perm)	Unique perms
20, 25	9.4604	0.0001	9284
20, 30	0.89937	0.3792	9307
25, 30	7.5617	0.0001	9293

Within level 'Pomace' of factor 'Subs'

Groups	t	P(perm)	Unique perms
20, 25	1.0394	0.3295	6628
20, 30	0.23.515	0.0027	508
25, 30	Negative		

Within level 'Red Onion' of factor 'Subs'

Groups	t	P(perm)	Unique perms
20, 25	4.8178	0.0003	9341

Within level 'Red Cabbage' of factor 'Subs'

Groups	t	P(perm)	Unique perms
20, 25	1.202	0.2358	9324

20, 30	4.2613	0.0008	9314
25, 30	9.971	0.0001	9342

20, 30	1.2968	0.2114	9342
25, 30	2.0763	0.0588	9374

Within level 'Spinach' of factor 'Subs'

Groups	t	P(perm)	Unique perms
20, 25	3.8002	0.0012	9377
20, 30	4.4168	0.0001	9337
25, 30	5.7057	0.0001	9358

PERMANOVA Bioconversion Ratio (BR)

**Factors**	**Abbrev.**	**Type**	**Levels**
Name	Su	Fixed	7
Subs	Te	Fixed	3
Temp			

PERMANOVA table of results

Source	df	SS	MS	Pseudo-F	P(perm)	perms
Su	6	0.31179	0.051964	174.13	0.0001	9944
Te	2	0.005928	0.002964	9.9323	0.0002	9949
SuxTe	12	0.0088511	0.00073759	2.4717	0.0057	9941
Res	189	0.056401	0.00029842			
Total	209	0.38297				

PAIR-WISE TESTS Term 'SuxTe' for pairs of levels of factor 'Subs'

Within level '20' of factor 'Temp'

Groups	t	P(perm)	Unique perms
CONTROL, Pumpkin	5.7115	0.0001	9321
CONTROL, Apple	0.16424	0.8732	9361
CONTROL, Pomace	4.5297	0.0001	6561
CONTROL, Red Onion	7.9793	0.0001	9356
CONTROL, Red Cabbage	6.8611	0.0001	9330
CONTROL, Spinach	3.035	0.0027	9378
Pumpkin, Apple	7.3374	0.0001	9383
Pumpkin, Pomace	10.75	0.0001	6646
Pumpkin, Red Onion	1.2512	0.2231	9300
Pumpkin, Red Cabbage	0.21483	0.8181	9274
Pumpkin. Spinach	9.5976	0.0001	9328
Apple, Pomace	29.137	0.0001	6489
Apple, Red Onion	11.318	0.0001	9316
Apple, Red Cabbage	9.8618	0.0001	9348
Apple, Spinach	23.317	0.0001	9200
Pomace, Red Onion	15.661	0.0001	6620
Pomace, Red Cabbage	14.298	0.0001	6625
Pomace, Spinach	14.258	0.0002	6553
Red Onion, Red Cabbage	1.2006	0.2447	9347
Red Onion, Spinach	14.221	0.0001	9366
Red Cabbage, Spinach	12.823	0.0001	9340

Within level '25' of factor 'Temp'

Groups	t	P(perm)	Unique perms
CONTROL, Pumpkin	2.2481	0.0409	9333
CONTROL, Apple	6.6941	0.0001	9331
CONTROL, Pomace	7.3127	0.0001	9339
CONTROL, Red Onion	3.6982	0.0021	9307
CONTROL, Red Cabbage	2.7707	0.0135	9329
CONTROL, Spinach	8.1187	0.0001	9361
Pumpkin, Apple	9.7697	0.0001	9379
Pumpkin, Pomace	9.8389	0.0001	9026
Pumpkin, Red Onion	1.4904	0.1543	9307
Pumpkin, Red Cabbage	1.1036	0.2856	9387
Pumpkin. Spinach	11.035	0.0001	9325
Apple, Pomace	2.529	0.0228	9277
Apple, Red Onion	11.495	0.0001	9286
Apple, Red Cabbage	7.5347	0.0002	9330
Apple, Spinach	3.083	0.0073	9332
Pomace, Red Onion	11.301	0.0001	9282
Pomace, Red Cabbage	8.1686	0.0001	9310
Pomace, Spinach	0.27539	0.7395	9224
Red Onion, Red Cabbage	0.015522	0.9932	9305
Red Onion, Spinach	12.654	0.0001	9268
Red Cabbage, Spinach	8.5545	0.0001	9382

Within level '30' of factor 'Temp'

Groups	t	P(perm)	Unique perms
CONTROL, Pumpkin	2.9715	0.0115	9343
CONTROL, Apple	4.847	0.0001	9256
CONTROL, Pomace	8.9744	0.0001	6565
CONTROL, Red Onion	4.3161	0.0003	9338
CONTROL, Red Cabbage	3.5414	0.002	9324
CONTROL, Spinach	8.8121	0.0001	9331
Pumpkin, Apple	8.792	0.0001	9191
Pumpkin, Pomace	12.942	0.0001	6681
Pumpkin, Red Onion	0.80681	0.4232	9335
Pumpkin, Red Cabbage	0.9983	0.3585	9321
Pumpkin. Spinach	12.793	0.0001	9378
Apple, Pomace	12.867	0.0002	6282
Apple, Red Onion	13.921	0.0001	9158
Apple, Red Cabbage	8.0076	0.0001	9032
Apple, Spinach	12.648	0.0001	9165
Pomace, Red Onion	20.412	0.0001	6627

Pomace, Red Cabbage	11.129	0.0001	6633
Pomace, Spinach	0.92304	0.3584	6621
Red Onion, Red Cabbage	0.45884	0.652	9367
Red Onion, Spinach	20.263	0.0001	9134
Red Cabbage, Spinach	11.006	0.0001	9231

PAIR-WISE TESTS **Term 'SuxTe' for pairs of levels of factor 'Temp'**

Within level 'CONTROL' of factor 'Subs'

Groups	t	P(perm)	Unique perms
20, 25	4.9205	0.0001	9315
20, 30	3.2688	0.0071	9319
25, 30	1.5263	0.1435	9377

Within level 'Pumpkin' of factor 'Subs'

Groups	t	P(perm)	Unique perms
20, 25	0.13941	0.8713	9254
20, 30	0.30945	0.7246	9232
25, 30	0.537	0.6322	9198

Within level 'Apple' of factor 'Subs'

Groups	t	P(perm)	Unique perms
20, 25	0.80115	0.437	9340
20, 30	0.70911	0.4847	9376
25, 30	1.1387	0.2549	9240

Within level 'Pomace' of factor 'Subs'

Groups	t	P(perm)	Unique perms
20, 25	3.4361	0.0004	6646
20, 30	0.89667	0.3831	507
25, 30	3.1803	0.0066	6617

Within level 'Red Onion' of factor 'Subs'

Groups	t	P(perm)	Unique perms
20, 25	0.06187	0.94	9296
20, 30	1.2924	0.2117	9301
25, 30	1.4517	0.1628	9312

Within level 'Red Cabbage' of factor 'Subs'

Groups	t	P(perm)	Unique perms
20, 25	0.9978	0.3274	9321
20, 30	0.4888	0.6269	9319
25, 30	0.48117	0.6362	9334

PERMANOVA Efficiency Converison of Digested Feed (ECD)

Factors

Name	Abbrev.	Type	Levels
Subs	Su	Fixed	7
Temp	Te	Fixed	3

PERMANOVA table of results

Source	df	SS	MS	Pseudo-F	P(perm)	Unique perms
Su	6	0.93528	0.15588	184.4	0.0001	9941
Te	2	0.12074	0.060372	71.419	0.0001	9960
SuxTe	12	0.04987	0.0041558	4.9163	0.0001	9937
Res	189	0.15976	0.00084532			
Total	209	1.2657				

PAIR-WISE TESTS **Term 'SuxTe' for pairs of levels of factor 'Subs'**

Within level '20' of factor 'Temp'

Groups	t	P(perm)	Unique perms
CONTROL, Pumpkin	7.8008	0.0001	9386
CONTROL, Apple	4.8995	0.0002	9324
CONTROL, Pomace	8.7044	0.0001	6575
CONTROL, Red Onion	2.514	0.0227	9325
CONTROL, Red Cabbage	1.8436	0.0841	9318
CONTROL, Spinach	7.5001	0.0003	9326
Pumpkin, Apple	20.502	0.0001	9230
Pumpkin, Pomace	26.131	0.0001	6432
Pumpkin, Red Onion	2.1925	0.0418	9313
Pumpkin, Red Cabbage	3.7515	0.0021	9341
Pumpkin. Spinach	24.466	0.0001	9183
Apple, Pomace	26.085	0.0003	6460
Apple, Red Onion	5.5681	0.0001	9372
Apple, Red Cabbage	5.5051	0.0001	9338
Apple, Spinach	19.31	0.0001	9293
Pomace, Red Onion	7.6586	0.0001	6582
Pomace, Red Cabbage	8.0533	0.0001	6559
Pomace, Spinach	12.977	0.0001	6605
Red Onion, Red Cabbage	0.80996	0.4227	9298
Red Onion, Spinach	6.9921	0.0001	9333
Red Cabbage, Spinach	7.2426	0.0001	9282

Within level '25' of factor 'Temp'

Groups	t	P(perm)	Unique perms
CONTROL, Pumpkin	7.7124	0.0001	9353
CONTROL, Apple	5.8597	0.0002	9287
CONTROL, Pomace	7.8763	0.0001	9325
CONTROL, Red Onion	5.3675	0.0001	9309
CONTROL, Red Cabbage	1.1429	0.2633	9275
CONTROL, Spinach	7.4547	0.0001	9269
Pumpkin, Apple	62.229	0.0001	8503
Pumpkin, Pomace	35.465	0.0001	8949
Pumpkin, Red Onion	3.3013	0.0047	9308
Pumpkin, Red Cabbage	4.7933	0.0001	9377
Pumpkin. Spinach	38.824	0.0001	8914
Apple, Pomace	5.9772	0.0001	9330
Apple, Red Onion	22.535	0.0001	9223
Apple, Red Cabbage	6.1914	0.0002	9338
Apple, Spinach	5.2619	0.0001	9332
Pomace, Red Onion	21.859	0.0001	9154
Pomace, Red Cabbage	7.9086	0.0001	9336
Pomace, Spinach	1.1207	0.2649	9256
Red Onion, Red Cabbage	3.1351	0.0084	9319
Red Onion, Spinach	22.167	0.0001	9208
Red Cabbage, Spinach	7.5382	0.0001	9349

Within level '30' of factor 'Temp'

Groups	t	P(perm)	Unique perms
CONTROL, Pumpkin	3.3413	0.0053	9309
CONTROL, Apple	4.9669	0.0001	9386
CONTROL, Pomace	9.3836	0.0001	6643
CONTROL, Red Onion	4.9232	0.0002	9328
CONTROL, Red Cabbage	2.874	0.0109	9308
CONTROL, Spinach	9.2661	0.0001	9335
Pumpkin, Apple	6.5983	0.0001	9339
Pumpkin, Pomace	9.056	0.0001	6578
Pumpkin, Red Onion	0.33287	0.7417	9326
Pumpkin, Red Cabbage	0.55964	0.5994	9288
Pumpkin. Spinach	8.984	0.0001	9385
Apple, Pomace	15.025	0.0001	6563
Apple, Red Onion	13.925	0.0001	9287
Apple, Red Cabbage	6.4113	0.0001	9346
Apple, Spinach	15.011	0.0001	9279
Pomace, Red Onion	19.866	0.0001	6568
Pomace, Red Cabbage	9.1113	0.0001	6599
Pomace, Spinach	0.7007	0.4829	6567
Red Onion, Red Cabbage	0.39154	0.6899	9305
Red Onion, Spinach	19.769	0.0001	9233
Red Cabbage, Spinach	9.0332	0.0001	9342

PAIR-WISE TESTS **Term 'SuxTe' for pairs of levels of factor 'Temp'**

Within level 'CONTROL' of factor 'Subs'

Groups	t	P(perm)	Unique perms
20, 25	3.0978	0.0065	9358
20, 30	0.80971	0.4326	9326
25, 30	3.9521	0.0012	9348

Within level 'Pumpkin' of factor 'Subs'

Groups	t	P(perm)	Unique perms
20, 25	9.2037	0.0001	9333
20, 30	3.1564	0.0083	9350
25, 30	8.1043	0.0001	9399

Within level 'Apple' of factor 'Subs'

Groups	t	P(perm)	Unique perms
20, 25	9.3128	0.0001	9292
20, 30	0.76969	0.458	9330
25, 30	8.2381	0.0001	9342

Within level 'Pomace' of factor 'Subs'

Groups	t	P(perm)	Unique perms
20, 25	3.4856	0.0008	6646
20, 30	0.86884	0.4051	507
25, 30	3.2355	0.0042	6631

Within level 'Red Onion' of factor 'Subs'

Groups	t	P(perm)	Unique perms
20, 25	4.3645	0.0001	9298
20, 30	0.6691	0.5016	9318
25, 30	10.354	0.0001	9297

Within level 'Red Cabbage' of factor 'Subs'

Groups	t	P(perm)	Unique perms
20, 25	1.9902	0.0638	9307
20, 30	0.027158	0.985	9329
25, 30	2.0718	0.0574	9327

Within level 'Spinach' of factor 'Subs'

Groups	t	P(perm)	Unique perms
20, 25	3.4186	0.0007	9383
20, 30	8.1871	0.0001	9333
25, 30	5.2484	0.0001	9340

PERMANOVA Growth Rate (GR)

Factors

Name	Abbrev.	Type	Levels
Subs	Su	Fixed	7
Temp	Te	Fixed	3

PERMANOVA table of results

Source	df	SS	MS	Pseudo-F	P(perm)	Unique perms
Su	6	9.89E-05	1.65E-05	254.8	0.0001	9942
Te	2	5.24E-06	2.62E-06	40.534	0.0001	9945
SuxTe	12	3.94E-06	3.28E-07	5.0709	0.0001	9922
Res	189	1.22E-05	6.47E-08			
Total	209	0.0001203				

PAIR-WISE TESTS **Term 'SuxTe' for pairs of levels of factor 'Subs'**

Within level '20' of factor 'Temp'

Groups	t	P(perm)	Unique perms
CONTROL, Pumpkin	2.8736	0.0103	9325
CONTROL, Apple	16.019	0.0001	9238
CONTROL, Pomace	19.457	0.0001	6469
CONTROL, Red Onion	2.4562	0.0243	9354
CONTROL, Red Cabbage	3.7876	0.0016	9348
CONTROL, Spinach	8.7488	0.0001	9339
Pumpkin, Apple	21.138	0.0001	9197
Pumpkin, Pomace	24.79	0.0001	6507
Pumpkin, Red Onion	6.0043	0.0001	9350
Pumpkin, Red Cabbage	7.7142	0.0001	9304
Pumpkin. Spinach	11.677	0.0001	9297
Apple, Pomace	31.146	0.0001	6426
Apple, Red Onion	20.044	0.0001	9200
Apple, Red Cabbage	24.776	0.0001	9202
Apple, Spinach	2.6672	0.0187	9362
Pomace, Red Onion	25.346	0.0001	6518
Pomace, Red Cabbage	32.109	0.0001	6451
Pomace, Spinach	5.7358	0.0001	6662
Red Onion, Red Cabbage	1.5662	0.1358	9317
Red Onion, Spinach	7.8819	0.0001	9336
Red Cabbage, Spinach	7.3629	0.0001	9319

Within level '25' of factor 'Temp'

Groups	t	P(perm)	Unique perms
CONTROL, Pumpkin	3.9861	0.0002	9351
CONTROL, Apple	22.592	0.0001	9105
CONTROL, Pomace	35.871	0.0001	8934
CONTROL, Red Onion	2.1059	0.0436	9390
CONTROL, Red Cabbage	4.0417	0.0001	9314
CONTROL, Spinach	9.2456	0.0001	9313
Pumpkin, Apple	10.534	0.0001	9387
Pumpkin, Pomace	12.702	0.0001	9330
Pumpkin, Red Onion	1.9957	0.0637	9348
Pumpkin, Red Cabbage	1.0931	0.2816	9243
Pumpkin. Spinach	8.1838	0.0001	9282
Apple, Pomace	8.0067	0.0001	9288
Apple, Red Onion	10.906	0.0001	9324
Apple, Red Cabbage	13.78	0.0001	9318
Apple, Spinach	2.7745	0.0086	9330
Pomace, Red Onion	13.932	0.0001	9323
Pomace, Red Cabbage	17.279	0.0002	9305
Pomace, Spinach	6.7153	0.0001	9301
Red Onion, Red Cabbage	1.1906	0.2405	9298
Red Onion, Spinach	7.5332	0.0001	9265
Red Cabbage, Spinach	9.4945	0.0001	9341

Within level '30' of factor 'Temp'

Groups	t	P(perm)	Unique perms
CONTROL, Pumpkin	2.7496	0.0163	9418
CONTROL, Apple	13.037	0.0001	9346
CONTROL, Pomace	18.771	0.0001	6539
CONTROL, Red Onion	4.3159	0.0007	9341
CONTROL, Red Cabbage	3.4759	0.0013	9293
CONTROL, Spinach	11.276	0.0002	9330
Pumpkin, Apple	8.0431	0.0001	9373
Pumpkin, Pomace	10.149	0.0001	6696
Pumpkin, Red Onion	0.0741	0.9426	9340
Pumpkin, Red Cabbage	1.3689	0.1873	9306
Pumpkin. Spinach	7.5668	0.0001	9414
Apple, Pomace	18.431	0.0001	6618
Apple, Red Onion	14.523	0.0001	9261
Apple, Red Cabbage	27.062	0.0001	9239
Apple, Spinach	1.9521	0.0669	9315
Pomace, Red Onion	18.529	0.0001	6551
Pomace, Red Cabbage	37.922	0.0001	6484
Pomace, Spinach	13.412	0.0001	6575
Red Onion, Red Cabbage	2.2486	0.0344	9334
Red Onion, Spinach	13.337	0.0002	9287
Red Cabbage, Spinach	21.894	0.0002	9274

PAIR-WISE TESTS **Term 'SuxTe' for pairs of levels of factor 'Temp'**

Within level 'CONTROL' of factor 'Subs'

Groups	t	P(perm)	Unique perms
20, 25	1.342	0.2019	9291
20, 30	0.38761	0.7036	9297
25, 30	0.79614	0.4416	9330

Within level 'Pumpkin' of factor 'Subs'

Groups	t	P(perm)	Unique perms
20, 25	2.6873	0.0171	9339
20, 30	1.6178	0.1251	9300
25, 30	0.4781	0.6437	9211

Within level 'Apple' of factor 'Subs'

Groups	t	P(perm)	Unique perms
20, 25	6.1028	0.0002	9298
20, 30	8.6134	0.0001	9277
25, 30	0.77489	0.4383	9356

Within level 'Pomace' of factor 'Subs'

Groups	t	P(perm)	Unique perms
20, 25	6.3364	0.0001	6503
20, 30	1.3383	0.1815	507
25, 30	5.2348	0.0001	6577

Within level 'Red Onion' of factor 'Subs'

Groups	t	P(perm)	Unique perms
20, 25	4.5818	0.0003	9331
20, 30	6.9856	0.0001	9341
25, 30	1.5945	0.1312	9314

Within level 'Red Cabbage' of factor 'Subs'

Groups	t	P(perm)	Unique perms
20, 25	7.7936	0.0001	9330
20, 30	11.531	0.0001	9268
25, 30	1.736	0.1102	9314

Within level 'Spinach' of factor 'Subs'

Groups	t	P(perm)	Unique perms
20, 25	2.1052	0.0493	9325
20, 30	0.5066	0.6193	9358
25, 30	2.2131	0.0382	9354

Table S4 - 3-way Permanova analysis and pair-wise tests for development and bioconversion variables (all substrates except pomace). Variables – Weight; Waste Reduction Index (WRI); Feed Conversion Ratio (FCR); Bioconversion Ratio (BR); (Efficiency of conversion of digested feed (ECD);

PERMANOVA — Weight

Factors

Name	Abbrev.	Type	Levels
Subs	Su	Fixed	6
Temp	Te	Fixed	3
RH	RH	Fixed	2

PAIR-WISE TESTS — **Term 'SuxTexRH' for pairs of levels of factor 'Subs'**

Within level '20' of factor 'Temp'
Within level '70' of factor 'RH'

Groups	t	P(perm)	Unique perms
CONTROL, Pumpkin	5.3415	0.0065	126
CONTROL, Apple	13.709	0.0077	126
CONTROL, Red Onion	7.2221	0.0078	126
CONTROL, Red Cabbage	7.0684	0.0083	126
CONTROL, Spinach	9.3434	0.009	126
Pumpkin, Apple	19.726	0.0091	126
Pumpkin, Red Onion	3.7598	0.015	126
Pumpkin, Red Cabbage	3.4519	0.0131	126
Pumpkin. Spinach	7.6498	0.0083	126
Apple, Red Onion	11.686	0.0074	126
Apple, Red Cabbage	14.427	0.0069	126
Apple, Spinach	7.2186	0.0091	126
Red Onion, Red Cabbage	0.61411	0.485	126
Red Onion, Spinach	3.4714	0.0228	126
Red Cabbage, Spinach	4.3796	0.0097	126

Within level '20' of factor 'Temp'
Within level 'N' of factor 'RH'

Groups	t	P(perm)	Unique perms
CONTROL, Pumpkin	3.5887	0.0072	126
CONTROL, Apple	9.6308	0.0082	126
CONTROL, Red Onion	6.4124	0.0084	126
CONTROL, Red Cabbage	5.2159	0.0082	126
CONTROL, Spinach	10.259	0.007	126
Pumpkin, Apple	18.258	0.0087	126
Pumpkin, Red Onion	6.9224	0.0089	126
Pumpkin, Red Cabbage	4.3289	0.0165	126
Pumpkin. Spinach	20.077	0.0086	126
Apple, Red Onion	10.745	0.0071	126
Apple, Red Cabbage	26.721	0.0077	126
Apple, Spinach	6.1473	0.0095	126
Red Onion, Red Cabbage	4.4228	0.0095	126
Red Onion, Spinach	12.89	0.0078	126
Red Cabbage, Spinach	29.71	0.0072	126

Within level '25' of factor 'Temp'
Within level '70' of factor 'RH'

Groups	t	P(perm)	Unique perms
CONTROL, Pumpkin	9.9616	0.0071	126
CONTROL, Apple	22.078	0.0081	126
CONTROL, Red Onion	4.8518	0.0073	126
CONTROL, Red Cabbage	12.306	0.0076	126
CONTROL, Spinach	24.408	0.008	126
Pumpkin, Apple	20.235	0.009	126
Pumpkin, Red Onion	3.3933	0.0159	126
Pumpkin, Red Cabbage	5.2215	0.0078	126
Pumpkin. Spinach	22.236	0.009	126
Apple, Red Onion	15.95	0.0086	126
Apple, Red Cabbage	12.468	0.0077	126
Apple, Spinach	6.0014	0.0083	126
Red Onion, Red Cabbage	6.3647	0.0086	126
Red Onion, Spinach	19.029	0.0088	126
Red Cabbage, Spinach	16.243	0.0078	126

Within level '25' of factor 'Temp'
Within level 'N' of factor 'RH'

Groups	t	P(perm)	Unique perms
CONTROL, Pumpkin	12.818	0.009	126
CONTROL, Apple	23.472	0.0082	126
CONTROL, Red Onion	16.428	0.0074	126
CONTROL, Red Cabbage	5.4807	0.0064	126
CONTROL, Spinach	5.6361	0.0074	125
Pumpkin, Apple	19.41	0.0075	126
Pumpkin, Red Onion	8.1738	0.0079	126
Pumpkin, Red Cabbage	14.106	0.0087	126
Pumpkin. Spinach	0.88333	0.4109	126
Apple, Red Onion	11.343	0.0086	126
Apple, Red Cabbage	27.407	0.0065	126
Apple, Spinach	4.8571	0.0085	126
Red Onion, Red Cabbage	18.512	0.0082	126
Red Onion, Spinach	1.0749	0.3174	126
Red Cabbage, Spinach	3.7189	0.008	126

Within level '30' of factor 'Temp'
Within level '70' of factor 'RH'

Groups	t	P(perm)	Unique perms
CONTROL, Pumpkin	5.5876	0.0086	126
CONTROL, Apple	19.101	0.0079	126
CONTROL, Red Onion	1.9368	0.0952	126
CONTROL, Red Cabbage	2.1818	0.0642	126
CONTROL, Spinach	16.688	0.0084	126
Pumpkin, Apple	16.137	0.0073	126
Pumpkin, Red Onion	2.659	0.0162	126
Pumpkin, Red Cabbage	1.8533	0.0536	126
Pumpkin. Spinach	13.562	0.0079	126
Apple, Red Onion	13.365	0.0083	126
Apple, Red Cabbage	10.931	0.0098	126
Apple, Spinach	4.3904	0.0081	126
Red Onion, Red Cabbage	0.41145	0.5498	126
Red Onion, Spinach	13.309	0.0082	126

Within level '30' of factor 'Temp'
Within level 'N' of factor 'RH'

Groups	t	P(perm)	Unique perms
CONTROL, Pumpkin	6.558	0.006	126
CONTROL, Apple	13.1	0.006	126
CONTROL, Red Onion	2.0177	0.0883	126
CONTROL, Red Cabbage	0.27298	0.8261	126
CONTROL, Spinach	10.173	0.0085	126
Pumpkin, Apple	15.481	0.0092	126
Pumpkin, Red Onion	2.085	0.0353	126
Pumpkin, Red Cabbage	17.343	0.0078	126
Pumpkin. Spinach	6.8353	0.0067	126
Apple, Red Onion	6.1799	0.0091	126
Apple, Red Cabbage	55.352	0.0071	126
Apple, Spinach	0.027644	0.9856	126
Red Onion, Red Cabbage	2.5677	0.0466	126
Red Onion, Spinach	5.4661	0.0088	126

Red Cabbage, Spinach	11.705	0.0086	126	Red Cabbage, Spinach	15.763	0.0086	126

PAIR-WISE TESTS **Term 'SuxTexRH' for pairs of levels of factor 'Temp'**

Within level 'CONTROL' of factor 'Subs'
Within level '70' of factor 'RH'

Groups	t	P(perm)	Unique perms
20, 25	1.5878	0.1289	126
20, 30	3.3959	0.0347	126
25, 30	4.2746	0.0079	125

Within level 'CONTROL' of factor 'Subs'
Within level 'N' of factor 'RH'

Groups	t	P(perm)	Unique perms
20, 25	1.0141	0.3217	126
20, 30	0.71195	0.4963	126
25, 30	2.9164	0.0075	126

Within level 'Pumpkin' of factor 'Subs'
Within level '70' of factor 'RH'

Groups	t	P(perm)	Unique perms
20, 25	2.1524	0.0597	126
20, 30	0.50497	0.6471	126
25, 30	3.8963	0.0088	126

Within level 'Pumpkin' of factor 'Subs'
Within level 'N' of factor 'RH'

Groups	t	P(perm)	Unique perms
20, 25	1.4227	0.2479	126
20, 30	2.5453	0.0516	126
25, 30	6.1278	0.0065	126

Within level 'Apple' of factor 'Subs'
Within level 'N' of factor 'RH'

Groups	t	P(perm)	Unique perms
20, 25	5.4518	0.0074	126
20, 30	13.194	0.0095	126
25, 30	0.38899	0.7075	126

Within level 'Red Onion' of factor 'Subs'
Within level 'N' of factor 'RH'

Groups	t	P(perm)	Unique perms
20, 25	5.16	0.0081	126
20, 30	4.1758	0.0089	126
25, 30	2.3844	0.0229	126

Within level 'Red Cabbage' of factor 'Subs'
Within level '70' of factor 'RH'

Groups	t	P(perm)	Unique perms
20, 25	3.4495	0.0257	126
20, 30	4.2296	0.0087	126
25, 30	2.1671	0.018	126

Within level 'Red Cabbage' of factor 'Subs'
Within level 'N' of factor 'RH'

Groups	t	P(perm)	Unique perms
20, 25	21.899	0.0082	126
20, 30	25.147	0.007	126
25, 30	1.1146	0.2923	126

Within level 'Spinach' of factor 'Subs'
Within level '70' of factor 'RH'

Groups	t	P(perm)	Unique perms
20, 25	2.541	0.0324	126
20, 30	3.5001	0.0082	126
25, 30	1.584	0.1241	126

Within level 'Spinach' of factor 'Subs'
Within level 'N' of factor 'RH'

Groups	t	P(perm)	Unique perms
20, 25	7.3432	0.0086	126
20, 30	4.3399	0.0075	126
25, 30	4.3254	0.0077	126

PAIR-WISE TESTS **Term 'SuxTexRH' for pairs of levels of factor 'RH'**

Within level 'CONTROL' of factor 'Subs'
Within level '20' of factor 'Temp'

Groups	t	P(perm)	Unique perms
70. N	1.4097	0.1942	126

Within level 'CONTROL' of factor 'Subs'
Within level '25' of factor 'Temp'

Groups	t	P(perm)	Unique perms
70. N	1.492	0.1856	126

Within level 'CONTROL' of factor 'Subs'
Within level '30' of factor 'Temp'

Groups	t	P(perm)	Unique perms
70. N	0.50691	0.621	126

Within level 'Pumpkin' of factor 'Subs'
Within level '20' of factor 'Temp'

Groups	t	P(perm)	Unique perms
70. N	1.9706	0.1005	126

Within level 'Pumpkin' of factor 'Subs'
Within level '25' of factor 'Temp'

Groups	t	P(perm)	Unique perms
70. N	7.6199	0.0081	126

Within level 'Pumpkin' of factor 'Subs'
Within level '30' of factor 'Temp'

Groups	t	P(perm)	Unique perms
70. N	5.6009	0.0094	126

Within level 'Apple' of factor 'Subs'
Within level '20' of factor 'Temp'

Groups	t	P(perm)	Unique perms
70. N	0.012173	1	125

Within level 'Apple' of factor 'Subs'
Within level '25' of factor 'Temp'

Groups	t	P(perm)	Unique perms
70. N	7.6326	0.0073	126

Within level 'Apple' of factor 'Subs'
Within level '30' of factor 'Temp'

Groups	t	P(perm)	Unique perms
70. N	9.1023	0.0072	126

Within level 'Red Onion' of factor 'Subs'
Within level '20' of factor 'Temp'

Groups	t	P(perm)	Unique perms
70. N	3.9126	0.0082	126

Within level 'Red Onion' of factor 'Subs'
Within level '25' of factor 'Temp'

Groups	t	P(perm)	Unique perms
70. N	12.015	0.0083	126

Within level 'Red Onion' of factor 'Subs'
Within level '30' of factor 'Temp'

Groups	t	P(perm)	Unique perms
70. N	0.95439	0.3727	126

Within level 'Red Cabbage' of factor 'Subs'
Within level '20' of factor 'Temp'

Groups	t	P(perm)	Unique perms
70. N	2.2392	0.0472	126

Within level 'Red Cabbage' of factor 'Subs'
Within level '25' of factor 'Temp'

Groups	t	P(perm)	Unique perms
70. N	10.923	0.007	126

Within level 'Red Cabbage' of factor 'Subs'
Within level '30' of factor 'Temp'

Groups	t	P(perm)	Unique perms
70. N	3.6566	0.0342	126

Within level 'Spinach' of factor 'Subs'
Within level '20' of factor 'Temp'

Groups	t	P(perm)	Unique perms
70. N	9.146	0.0076	126

Within level 'Spinach' of factor 'Subs'
Within level '25' of factor 'Temp'

Groups	t	P(perm)	Unique perms
70. N	4.3139	0.0066	126

Within level 'Spinach' of factor 'Subs'
Within level '30' of factor 'Temp'

Groups	t	P(perm)	Unique perms
70. N	0.44926	0.6569	126

PERMANOVA **Waste Reduction Index (WRI)**

Factors

**Name**	**Abbrev.**	**Type**	**Levels**
Subs	Su	Fixed	6
Temp	Te	Fixed	3
RH	RH	Fixed	2

PERMANOVA table of results

Source	df	SS	MS	Pseudo-F	P(perm)	Unique perms
Su	5	24.8	4.9599	2713.7	0.0001	9946
Te	2	1.2387	0.61936	338.86	0.0001	9947
RH	1	0.41164	0.41164	225.22	0.0001	9846
SuxTe	10	0.81481	0.081481	44.579	0.0001	9932
SuxRH	5	0.785	0.157	85.897	0.0001	9952
TexRH	2	0.5256	0.2628	143.78	0.0001	9948
SuxTexRH	10	0.85956	0.085956	47.028	0.0001	9933
Res	144	0.2632	0.0018278			
Total	179	29.698				

PAIR-WISE TESTS **Term 'SuxTexRH' for pairs of levels of factor 'Subs'**

Within level '20' of factor 'Temp'
Within level '70' of factor 'RH'

Groups	t	P(perm)	Unique perms
CONTROL, Pumpkin	11.076	0.0087	126
CONTROL, Apple	3.339	0.0235	126
CONTROL, Red Onion	41.319	0.0069	123
CONTROL, Red Cabbage	9.404	0.0083	126
CONTROL, Spinach	2.6082	0.0373	126
Pumpkin, Apple	12.134	0.0087	126
Pumpkin, Red Onion	1.4806	0.2509	126
Pumpkin, Red Cabbage	7.0881	0.0084	126
Pumpkin. Spinach	7.4076	0.0073	126
Apple, Red Onion	44.905	0.0094	126
Apple, Red Cabbage	11.985	0.0095	126
Apple, Spinach	3.9893	0.0157	126
Red Onion, Red Cabbage	17.281	0.006	126
Red Onion, Spinach	10.264	0.008	126

Within level '20' of factor 'Temp'
Within level 'N' of factor 'RH'

Groups	t	P(perm)	Unique perms
CONTROL, Pumpkin	49.896	0.0077	126
CONTROL, Apple	3.1049	0.0318	126
CONTROL, Red Onion	59.008	0.0068	126
CONTROL, Red Cabbage	27.21	0.0075	126
CONTROL, Spinach	0.62881	0.5168	126
Pumpkin, Apple	96.893	0.0096	126
Pumpkin, Red Onion	18.34	0.0088	126
Pumpkin, Red Cabbage	29.263	0.0073	126
Pumpkin. Spinach	51.915	0.0087	126
Apple, Red Onion	102.32	0.0084	126
Apple, Red Cabbage	41.695	0.0087	126
Apple, Spinach	4.0503	0.0157	126
Red Onion, Red Cabbage	42.272	0.0084	126
Red Onion, Spinach	61.08	0.0085	126

Red Cabbage, Spinach	2.1725	0.0341	126	Red Cabbage, Spinach	28.525	0.0085	126

Within level '25' of factor 'Temp'
Within level '70' of factor 'RH'

Groups	t	P(perm)	Unique perms
CONTROL, Pumpkin	84.335	0.0074	126
CONTROL, Apple	39.654	0.0076	126
CONTROL, Red Onion	25.44	0.0076	126
CONTROL, Red Cabbage	36.393	0.0078	126
CONTROL, Spinach	1.8624	0.0473	126
Pumpkin, Apple	98.861	0.0081	126
Pumpkin, Red Onion	7.5328	0.008	126
Pumpkin, Red Cabbage	3.5356	0.0351	126
Pumpkin. Spinach	32.276	0.0066	126
Apple, Red Onion	31.801	0.0077	126
Apple, Red Cabbage	42.569	0.0081	126
Apple, Spinach	4.3728	0.0081	126
Red Onion, Red Cabbage	8.3398	0.0073	126
Red Onion, Spinach	19.134	0.0084	126
Red Cabbage, Spinach	27.104	0.0083	126

Within level '25' of factor 'Temp'
Within level 'N' of factor 'RH'

Groups	t	P(perm)	Unique perms
CONTROL, Pumpkin	99.788	0.0079	126
CONTROL, Apple	3.9856	0.0079	126
CONTROL, Red Onion	142.3	0.0085	126
CONTROL, Red Cabbage	77.264	0.007	126
CONTROL, Spinach	5.7621	0.0079	126
Pumpkin, Apple	104.98	0.0077	126
Pumpkin, Red Onion	48.462	0.0082	126
Pumpkin, Red Cabbage	59.867	0.0077	126
Pumpkin. Spinach	50.097	0.0091	126
Apple, Red Onion	148.33	0.007	126
Apple, Red Cabbage	83.159	0.0071	126
Apple, Spinach	8.309	0.0094	126
Red Onion, Red Cabbage	197.35	0.008	126
Red Onion, Spinach	67.892	0.0068	126
Red Cabbage, Spinach	33.198	0.0067	126

Within level '30' of factor 'Temp'
Within level '70' of factor 'RH'

Groups	t	P(perm)	Unique perms
CONTROL, Pumpkin	27.25	0.0074	126
CONTROL, Apple	19.439	0.0063	126
CONTROL, Red Onion	21.922	0.0073	126
CONTROL, Red Cabbage	10.058	0.0072	126
CONTROL, Spinach	4.6618	0.008	126
Pumpkin, Apple	59.523	0.0093	126
Pumpkin, Red Onion	8.1693	0.0078	126
Pumpkin, Red Cabbage	22.283	0.0082	126
Pumpkin. Spinach	25.146	0.0084	126
Apple, Red Onion	60.623	0.007	126
Apple, Red Cabbage	47.804	0.0082	126
Apple, Spinach	6.8907	0.0068	126
Red Onion, Red Cabbage	15.599	0.008	126
Red Onion, Spinach	20.716	0.008	126
Red Cabbage, Spinach	12.132	0.0077	126

Within level '30' of factor 'Temp'
Within level 'N' of factor 'RH'

Groups	t	P(perm)	Unique perms
CONTROL, Pumpkin	61.059	0.0082	126
CONTROL, Apple	2.033	0.0808	126
CONTROL, Red Onion	79.943	0.0089	126
CONTROL, Red Cabbage	54.44	0.0086	126
CONTROL, Spinach	8.1362	0.0071	126
Pumpkin, Apple	84.715	0.0078	126
Pumpkin, Red Onion	23.196	0.0095	126
Pumpkin, Red Cabbage	13.817	0.0084	126
Pumpkin. Spinach	72.764	0.0071	126
Apple, Red Onion	107.48	0.008	126
Apple, Red Cabbage	82.401	0.008	126
Apple, Spinach	8.3354	0.0076	126
Red Onion, Red Cabbage	38.799	0.0062	126
Red Onion, Spinach	91.951	0.0092	126
Red Cabbage, Spinach	67.196	0.0073	126

PAIR-WISE TESTS **Term 'SuxTexRH' for pairs of levels of factor 'Temp'**

Within level 'CONTROL' of factor 'Subs'
Within level '70' of factor 'RH'

Groups	t	P(perm)	Unique perms
20, 25	12.347	0.007	126
20, 30	16.293	0.0083	126
25, 30	10.999	0.0081	126

Within level 'CONTROL' of factor 'Subs'
Within level 'N' of factor 'RH'

Groups	t	P(perm)	Unique perms
20, 25	4.0139	0.0161	125
20, 30	6.0793	0.0083	126
25, 30	3.5742	0.0162	126

Within level 'Pumpkin' of factor 'Subs'
Within level '70' of factor 'RH'

Groups	t	P(perm)	Unique perms
20, 25	4.2084	0.0099	126
20, 30	6.9182	0.0078	126
25, 30	8.7746	0.0067	126

Within level 'Pumpkin' of factor 'Subs'
Within level 'N' of factor 'RH'

Groups	t	P(perm)	Unique perms
20, 25	4.5371	0.0067	126
20, 30	1.7303	0.1524	126
25, 30	6.3217	0.0071	126

Within level 'Apple' of factor 'Subs'
Within level '70' of factor 'RH'

Groups	t	P(perm)	Unique perms
20, 25	6.3666	0.0087	126
20, 30	5.1299	0.0081	126
25, 30	4.6423	0.008	126

Within level 'Apple' of factor 'Subs'
Within level 'N' of factor 'RH'

Groups	t	P(perm)	Unique perms
20, 25	2.5517	0.0552	126
20, 30	5.8882	0.0086	126
25, 30	7.1255	0.0079	126

Within level 'Red Onion' of factor 'Subs'
Within level '70' of factor 'RH'

Within level 'Red Onion' of factor 'Subs'
Within level 'N' of factor 'RH'

Groups	t	P(perm)	Unique perms
20, 25	6.9866	0.0081	126
20, 30	21.488	0.0082	126
25, 30	6.2251	0.0096	126

Groups	t	P(perm)	Unique perms
20, 25	6.9042	0.0104	126
20, 30	2.8574	0.0153	126
25, 30	3.3476	0.0256	126

Within level 'Red Cabbage' of factor 'Subs'
Within level '70' of factor 'RH'

Groups	t	P(perm)	Unique perms
20, 25	25.543	0.0081	126
20, 30	16.113	0.0088	126
25, 30	15.333	0.0088	126

Within level 'Red Cabbage' of factor 'Subs'
Within level 'N' of factor 'RH'

Groups	t	P(perm)	Unique perms
20, 25	12.337	0.0069	126
20, 30	17.747	0.0076	126
25, 30	13.432	0.0074	126

Within level 'Spinach' of factor 'Subs'
Within level '70' of factor 'RH'

Groups	t	P(perm)	Unique perms
20, 25	0.10118	0.9275	126
20, 30	1.7329	0.1147	126
25, 30	2.3322	0.0494	126

Within level 'Spinach' of factor 'Subs'
Within level 'N' of factor 'RH'

Groups	t	P(perm)	Unique perms
20, 25	8.3691	0.0069	126
20, 30	1.234	0.27	126
25, 30	9.2267	0.0079	126

PAIR-WISE TESTS **Term 'SuxTexRH' for pairs of levels of factor 'RH'**

Within level 'CONTROL' of factor 'Subs'
Within level '20' of factor 'Temp'

Groups	t	P(perm)	Unique perms
70. N	1.5797	0.1666	126

Within level 'CONTROL' of factor 'Subs'
Within level '25' of factor 'Temp'

Groups	t	P(perm)	Unique perms
70. N	22.918	0.0068	126

Within level 'CONTROL' of factor 'Subs'
Within level '30' of factor 'Temp'

Groups	t	P(perm)	Unique perms
70. N	15.604	0.0098	126

Within level 'Pumpkin' of factor 'Subs'
Within level '20' of factor 'Temp'

Groups	t	P(perm)	Unique perms
70. N	0.02206	0.9603	126

Within level 'Pumpkin' of factor 'Subs'
Within level '25' of factor 'Temp'

Groups	t	P(perm)	Unique perms
70. N	23.664	0.0078	126

Within level 'Pumpkin' of factor 'Subs'
Within level '30' of factor 'Temp'

Groups	t	P(perm)	Unique perms
70. N	23.001	0.0083	126

Within level 'Apple' of factor 'Subs'
Within level '20' of factor 'Temp'

Groups	t	P(perm)	Unique perms
70. N	5.3393	0.0071	126

Within level 'Apple' of factor 'Subs'
Within level '25' of factor 'Temp'

Groups	t	P(perm)	Unique perms
70. N	4.8233	0.0092	126

Within level 'Apple' of factor 'Subs'
Within level '30' of factor 'Temp'

Groups	t	P(perm)	Unique perms
70. N	7.8127	0.0085	126

Within level 'Red Onion' of factor 'Subs'
Within level '20' of factor 'Temp'

Groups	t	P(perm)	Unique perms
70. N	31.698	0.0066	126

Within level 'Red Onion' of factor 'Subs'
Within level '25' of factor 'Temp'

Groups	t	P(perm)	Unique perms
70. N	6.6157	0.0086	126

Within level 'Red Onion' of factor 'Subs'
Within level '30' of factor 'Temp'

Groups	t	P(perm)	Unique perms
70. N	2.5457	0.0248	126

Within level 'Red Cabbage' of factor 'Subs'
Within level '20' of factor 'Temp'

Groups	t	P(perm)	Unique perms
70. N	7.1432	0.0071	126

Within level 'Red Cabbage' of factor 'Subs'
Within level '25' of factor 'Temp'

Groups	t	P(perm)	Unique perms
70. N	22.843	0.0079	126

Within level 'Red Cabbage' of factor 'Subs'
Within level '30' of factor 'Temp'

Groups	t	P(perm)	Unique perms

Within level 'Spinach' of factor 'Subs'
Within level '20' of factor 'Temp'

Groups	t	P(perm)	Unique perms

70. N	4.4796	0.008	126	70. N	3.5046	0.0231	126

Within level 'Spinach' of factor 'Subs'
Within level '25' of factor 'Temp'

Groups	t	P(perm)	Unique perms
70. N	1.0038	0.4092	126

Within level 'Spinach' of factor 'Subs'
Within level '30' of factor 'Temp'

Groups	t	P(perm)	Unique perms
70. N	7.7312	0.0081	126

PERMANOVA Feed Conversion Ratio

Factors

**Name**	**Abbrev.**	**Type**	**Levels**
Subs	Su	Fixed	6
Temp	Te	Fixed	3
RH	RH	Fixed	2

PERMANOVA table of results

Source	df	SS	MS	Pseudo-F	P(perm)	Unique perms
Su	5	156.44	31.289	680.36	0.0001	9952
Te	2	16.642	8.3212	180.94	0.0001	9944
RH	1	0.43542	0.43542	9.4679	0.0011	9831
SuxTe	10	13.673	1.3673	29.73	0.0001	9950
SuxRH	5	9.0069	1.8014	39.17	0.0001	9946
TexRH	2	0.068457	0.034229	0.74428	0.4926	9954
SuxTexRH	10	3.9695	0.39695	8.6315	0.0001	9937
Res	144	6.6224	0.045989			
Total	179	206.86				

PAIR-WISE TESTS **Term 'SuxTexRH' for pairs of levels of factor 'Subs'**

Within level '20' of factor 'Temp'
Within level '70' of factor 'RH'

Groups	t	P(perm)	Unique perms
CONTROL, Pumpkin	11.412	0.0069	126
CONTROL, Apple	9.1432	0.0076	126
CONTROL, Red Onion	10.61	0.008	126
CONTROL, Red Cabbage	8.3509	0.0066	126
CONTROL, Spinach	23.174	0.0083	126
Pumpkin, Apple	29.2	0.008	126
Pumpkin, Red Onion	0.63021	0.5528	126
Pumpkin, Red Cabbage	3.5103	0.0236	125
Pumpkin. Spinach	47.55	0.0083	126
Apple, Red Onion	26.825	0.0072	126
Apple, Red Cabbage	22.912	0.0075	126
Apple, Spinach	18.077	0.0077	126
Red Onion, Red Cabbage	2.7293	0.0312	126
Red Onion, Spinach	44.437	0.0075	126
Red Cabbage, Spinach	40.025	0.0071	126

Within level '20' of factor 'Temp'
Within level 'N' of factor 'RH'

Groups	t	P(perm)	Unique perms
CONTROL, Pumpkin	2.2092	0.0064	126
CONTROL, Apple	2.258	0.0452	126
CONTROL, Red Onion	1.9019	0.0158	126
CONTROL, Red Cabbage	1.115	0.3405	126
CONTROL, Spinach	5.2055	0.0064	126
Pumpkin, Apple	37.584	0.0082	126
Pumpkin, Red Onion	2.762	0.0231	126
Pumpkin, Red Cabbage	12.45	0.0083	126
Pumpkin. Spinach	36.853	0.0096	126
Apple, Red Onion	35.697	0.0088	126
Apple, Red Cabbage	35.667	0.0086	126
Apple, Spinach	14.654	0.0094	126
Red Onion, Red Cabbage	9.2924	0.0091	126
Red Onion, Spinach	35.576	0.0068	126
Red Cabbage, Spinach	33.644	0.0065	126

Within level '25' of factor 'Temp'
Within level '70' of factor 'RH'

Groups	t	P(perm)	Unique perms
CONTROL, Pumpkin	12.59	0.0072	126
CONTROL, Apple	37.799	0.0066	126
CONTROL, Red Onion	5.8058	0.007	126
CONTROL, Red Cabbage	5.2548	0.007	126
CONTROL, Spinach	29.113	0.0086	126
Pumpkin, Apple	68.8	0.0081	126
Pumpkin, Red Onion	1.0278	0.408	126
Pumpkin, Red Cabbage	4.8554	0.0096	126
Pumpkin. Spinach	33.602	0.0089	126
Apple, Red Onion	31.004	0.008	126
Apple, Red Cabbage	41.001	0.0083	126
Apple, Spinach	16.781	0.0078	126
Red Onion, Red Cabbage	1.8755	0.0846	126
Red Onion, Spinach	30.092	0.0086	126
Red Cabbage, Spinach	30.903	0.008	126

Within level '25' of factor 'Temp'
Within level 'N' of factor 'RH'

Groups	t	P(perm)	Unique perms
CONTROL, Pumpkin	8.2946	0.0081	126
CONTROL, Apple	5.0066	0.0076	126
CONTROL, Red Onion	6.8016	0.0085	126
CONTROL, Red Cabbage	1.3757	0.1789	126
CONTROL, Spinach	6.8538	0.0079	126
Pumpkin, Apple	25.083	0.0077	126
Pumpkin, Red Onion	6.7242	0.0081	126
Pumpkin, Red Cabbage	38.141	0.0089	126
Pumpkin. Spinach	28.43	0.0069	126
Apple, Red Onion	22.34	0.0067	126
Apple, Red Cabbage	12.895	0.0078	126
Apple, Spinach	2.7666	0.0311	126
Red Onion, Red Cabbage	29.201	0.0075	126
Red Onion, Spinach	25.721	0.0085	126
Red Cabbage, Spinach	16.506	0.0085	126

Within level '30' of factor 'Temp'
Within level '70' of factor 'RH'

Groups	t	P(perm)	Unique perms
CONTROL, Pumpkin	15.607	0.0079	126
CONTROL, Apple	26.414	0.0086	126
CONTROL, Red Onion	7.986	0.0084	126
CONTROL, Red Cabbage	9.4516	0.0083	126
CONTROL, Spinach	9.9731	0.0071	126
Pumpkin, Apple	59.054	0.0072	126
Pumpkin, Red Onion	4.9745	0.0086	126
Pumpkin, Red Cabbage	2.1646	0.0888	126
Pumpkin. Spinach	11.143	0.0067	126
Apple, Red Onion	33.748	0.0065	126
Apple, Red Cabbage	32.719	0.0092	126
Apple, Spinach	8.1092	0.0069	126
Red Onion, Red Cabbage	1.9508	0.0987	126
Red Onion, Spinach	10.722	0.0089	126
Red Cabbage, Spinach	10.922	0.0072	126

Within level '30' of factor 'Temp'
Within level 'N' of factor 'RH'

Groups	t	P(perm)	Unique perms
CONTROL, Pumpkin	7.2621	0.0085	126
CONTROL, Apple	8.5544	0.0094	126
CONTROL, Red Onion	10.674	0.0084	126
CONTROL, Red Cabbage	6.8335	0.0101	126
CONTROL, Spinach	13.662	0.0083	126
Pumpkin, Apple	29.247	0.0088	126
Pumpkin, Red Onion	5.4225	0.0067	126
Pumpkin, Red Cabbage	2.1644	0.0858	126
Pumpkin. Spinach	17.849	0.0082	126
Apple, Red Onion	42.386	0.0079	126
Apple, Red Cabbage	42.674	0.007	126
Apple, Spinach	10.935	0.0077	126
Red Onion, Red Cabbage	11.004	0.0092	126
Red Onion, Spinach	19.37	0.0109	126
Red Cabbage, Spinach	17.691	0.0077	126

PAIR-WISE TESTS **Term 'SuxTexRH' for pairs of levels of factor 'Temp'**

Within level 'CONTROL' of factor 'Subs'
Within level '70' of factor 'RH'

Groups	t	P(perm)	Unique perms
20, 25	10.636	0.007	126
20, 30	2.532	0.0528	126
25, 30	15.459	0.0081	126

Within level 'Pumpkin' of factor 'Subs'
Within level '70' of factor 'RH'

Groups	t	P(perm)	Unique perms
20, 25	6.3359	0.0085	126
20, 30	3.3597	0.0172	126
25, 30	16.056	0.0083	126

Within level 'Apple' of factor 'Subs'
Within level '70' of factor 'RH'

Groups	t	P(perm)	Unique perms
20, 25	8.8634	0.0081	126
20, 30	1.0001	0.3315	126
25, 30	16.012	0.0082	126

Within level 'Red Onion' of factor 'Subs'
Within level '70' of factor 'RH'

Groups	t	P(perm)	Unique perms
20, 25	3.6461	0.0084	126
20, 30	5.5978	0.0082	126
25, 30	9.3169	0.0089	126

Within level 'Red Cabbage' of factor 'Subs'
Within level '70' of factor 'RH'

Groups	t	P(perm)	Unique perms
20, 25	5.4133	0.008	126
20, 30	0.49465	0.7031	126
25, 30	6.5192	0.0065	126

Within level 'Spinach' of factor 'Subs'
Within level '70' of factor 'RH'

Groups	t	P(perm)	Unique perms
20, 25	2.1696	0.0704	126
20, 30	5.4116	0.0072	126
25, 30	5.7989	0.0077	126

Within level 'CONTROL' of factor 'Subs'
Within level 'N' of factor 'RH'

Groups	t	P(perm)	Unique perms
20, 25	0.73206	0.6868	126
20, 30	1.0407	0.3476	126
25, 30	5.0801	0.0087	126

Within level 'Pumpkin' of factor 'Subs'
Within level 'N' of factor 'RH'

Groups	t	P(perm)	Unique perms
20, 25	11.958	0.0087	126
20, 30	14.517	0.0082	126
25, 30	28.152	0.0085	126

Within level 'Apple' of factor 'Subs'
Within level 'N' of factor 'RH'

Groups	t	P(perm)	Unique perms
20, 25	6.8885	0.0068	126
20, 30	4.467	0.0082	126
25, 30	10.027	0.0092	126

Within level 'Red Onion' of factor 'Subs'
Within level 'N' of factor 'RH'

Groups	t	P(perm)	Unique perms
20, 25	10.569	0.0091	126
20, 30	8.3361	0.007	126
25, 30	22.053	0.0073	126

Within level 'Red Cabbage' of factor 'Subs'
Within level 'N' of factor 'RH'

Groups	t	P(perm)	Unique perms
20, 25	1.5689	0.1545	126
20, 30	17.547	0.0083	126
25, 30	20.707	0.0078	126

Within level 'Spinach' of factor 'Subs'
Within level 'N' of factor 'RH'

Groups	t	P(perm)	Unique perms
20, 25	14.573	0.0085	126
20, 30	4.892	0.0082	126
25, 30	12.667	0.0076	126

PAIR-WISE TESTS **Term 'SuxTexRH' for pairs of levels of factor 'RH'**

Within level 'CONTROL' of factor 'Subs'
Within level '20' of factor 'Temp'

Groups	t	P(perm)	Unique perms
70. N	0.23763	0.949	126

Within level 'CONTROL' of factor 'Subs'
Within level '25' of factor 'Temp'

Groups	t	P(perm)	Unique perms
70. N	5.3985	0.008	126

Within level 'CONTROL' of factor 'Subs'
Within level '30' of factor 'Temp'

Groups	t	P(perm)	Unique perms
70. N	8.9588	0.0072	126

Within level 'Pumpkin' of factor 'Subs'
Within level '20' of factor 'Temp'

Groups	t	P(perm)	Unique perms
70. N	4.2141	0.0077	126

Within level 'Pumpkin' of factor 'Subs'
Within level '25' of factor 'Temp'

Groups	t	P(perm)	Unique perms
70. N	0.10854	0.9292	126

Within level 'Pumpkin' of factor 'Subs'
Within level '30' of factor 'Temp'

Groups	t	P(perm)	Unique perms
70. N	18.212	0.0087	126

Within level 'Apple' of factor 'Subs'
Within level '20' of factor 'Temp'

Groups	t	P(perm)	Unique perms
70. N	2.2008	0.0645	126

Within level 'Apple' of factor 'Subs'
Within level '25' of factor 'Temp'

Groups	t	P(perm)	Unique perms
70. N	1.7057	0.1448	126

Within level 'Apple' of factor 'Subs'
Within level '30' of factor 'Temp'

Groups	t	P(perm)	Unique perms
70. N	12.635	0.0072	126

Within level 'Red Onion' of factor 'Subs'
Within level '20' of factor 'Temp'

Groups	t	P(perm)	Unique perms
70. N	5.4745	0.0083	126

Within level 'Red Onion' of factor 'Subs'
Within level '25' of factor 'Temp'

Groups	t	P(perm)	Unique perms
70. N	2.7671	0.0363	126

Within level 'Red Onion' of factor 'Subs'
Within level '30' of factor 'Temp'

Groups	t	P(perm)	Unique perms
70. N	6.5116	0.0081	126

Within level 'Red Cabbage' of factor 'Subs'
Within level '20' of factor 'Temp'

Groups	t	P(perm)	Unique perms
70. N	8.3397	0.0097	126

Within level 'Red Cabbage' of factor 'Subs'
Within level '25' of factor 'Temp'

Groups	t	P(perm)	Unique perms
70. N	22.677	0.0076	126

Within level 'Red Cabbage' of factor 'Subs'
Within level '30' of factor 'Temp'

Groups	t	P(perm)	Unique perms
70. N	16.194	0.0073	126

Within level 'Spinach' of factor 'Subs'
Within level '20' of factor 'Temp'

Groups	t	P(perm)	Unique perms
70. N	0.6146	0.4965	126

Within level 'Spinach' of factor 'Subs'
Within level '25' of factor 'Temp'

Groups	t	P(perm)	Unique perms
70. N	10.869	0.01	126

Within level 'Spinach' of factor 'Subs'
Within level '30' of factor 'Temp'

Groups	t	P(perm)	Unique perms
70. N	3.6345	0.0076	126

PERMANOVA Bioconversion Ratio (BR)

Factors

**Name**	**Abbrev.**	**Type**	**Levels**
Subs	Su	Fixed	6
Temp	Te	Fixed	3
RH	RH	Fixed	2

PERMANOVA table of results

Source	df	SS	MS	Pseudo-F	P(perm)	Unique perms
Su	5	0.22512	0.045024	1844.1	0.0001	9961
Te	2	0.0043348	0.0021674	88.774	0.0001	9960
RH	1	0.031387	0.031387	1285.6	0.0001	9843
SuxTe	10	0.0086218	0.00086218	35.314	0.0001	9934

SuxRH	5	0.017321	0.0034642	141.89	0.0001	9938
TexRH	2	4.05E-06	2.02E-06	0.082915	0.9224	9957
SuxTexRH	10	0.0018664	0.00018664	7.6446	0.0001	9930
Res	144	0.0035157	2.44E-05			
Total	179	0.29217				

PAIR-WISE TESTS **Term 'SuxTexRH' for pairs of levels of factor 'Subs'**

Within level '20' of factor 'Temp'
Within level '70' of factor 'RH'

Groups	t	P(perm)	Unique perms
CONTROL, Pumpkin	11.447	0.0082	126
CONTROL, Apple	4.4418	0.0068	126
CONTROL, Red Onion	12.828	0.008	126
CONTROL, Red Cabbage	8.82	0.0078	126
CONTROL, Spinach	9.1971	0.0085	126
Pumpkin, Apple	19.604	0.0077	126
Pumpkin, Red Onion	1.3322	0.2191	126
Pumpkin, Red Cabbage	0.65725	0.536	126
Pumpkin. Spinach	25.337	0.0074	126
Apple, Red Onion	21.525	0.0081	126
Apple, Red Cabbage	13.819	0.0077	126
Apple, Spinach	10.991	0.0082	126
Red Onion, Red Cabbage	1.7593	0.1039	126
Red Onion, Spinach	27.443	0.0078	126
Red Cabbage, Spinach	17.544	0.0087	126

Within level '20' of factor 'Temp'
Within level 'N' of factor 'RH'

Groups	t	P(perm)	Unique perms
CONTROL, Pumpkin	68.47	0.0075	126
CONTROL, Apple	21.797	0.0087	126
CONTROL, Red Onion	42.999	0.007	126
CONTROL, Red Cabbage	79.258	0.0099	126
CONTROL, Spinach	1.7091	0.0075	126
Pumpkin, Apple	1009.3	0.0006	146
Pumpkin, Red Onion	12.507	0.0075	126
Pumpkin, Red Cabbage	86.005	0.0074	126
Pumpkin. Spinach	1391.2	0.0073	152
Apple, Red Onion	37.104	0.008	126
Apple, Red Cabbage	432.71	0.0087	128
Apple, Spinach	433.05	0.0082	119
Red Onion, Red Cabbage	6.4759	0.009	126
Red Onion, Spinach	49.493	0.0094	126
Red Cabbage, Spinach	601.11	0.0036	133

Within level '25' of factor 'Temp'
Within level '70' of factor 'RH'

Groups	t	P(perm)	Unique perms
CONTROL, Pumpkin	5.9783	0.0075	126
CONTROL, Apple	18.983	0.008	126
CONTROL, Red Onion	8.0797	0.0079	126
CONTROL, Red Cabbage	11.33	0.0068	126
CONTROL, Spinach	28.208	0.0087	126
Pumpkin, Apple	42.325	0.0077	126
Pumpkin, Red Onion	3.9255	0.0162	126
Pumpkin, Red Cabbage	7.7611	0.0087	126
Pumpkin. Spinach	54.368	0.0065	126
Apple, Red Onion	29.636	0.0069	126
Apple, Red Cabbage	36.788	0.007	126
Apple, Spinach	26.464	0.0073	126
Red Onion, Red Cabbage	2.7993	0.0205	126
Red Onion, Spinach	38.363	0.0082	126
Red Cabbage, Spinach	46.004	0.0079	126

Within level '25' of factor 'Temp'
Within level 'N' of factor 'RH'

Groups	t	P(perm)	Unique perms
CONTROL, Pumpkin	9.7996	0.0095	126
CONTROL, Apple	17.041	0.0071	126
CONTROL, Red Onion	16.31	0.0072	126
CONTROL, Red Cabbage	10.54	0.0082	126
CONTROL, Spinach	14.832	0.0083	126
Pumpkin, Apple	903.53	0.0065	151
Pumpkin, Red Onion	231.26	0.0084	126
Pumpkin, Red Cabbage	15.355	0.007	126
Pumpkin. Spinach	1382.6	0.0056	155
Apple, Red Onion	877.8	0.0054	142
Apple, Red Cabbage	499.94	0.0075	129
Apple, Spinach	71.133	0.0075	126
Red Onion, Red Cabbage	105.97	0.0081	126
Red Onion, Spinach	1050.6	0.0081	149
Red Cabbage, Spinach	509.54	0.0072	129

Within level '30' of factor 'Temp'
Within level '70' of factor 'RH'

Groups	t	P(perm)	Unique perms
CONTROL, Pumpkin	11.199	0.0085	126
CONTROL, Apple	24.487	0.0079	126
CONTROL, Red Onion	6.9759	0.0097	126
CONTROL, Red Cabbage	6.8263	0.008	126
CONTROL, Spinach	41.62	0.0085	126
Pumpkin, Apple	40.581	0.0067	126
Pumpkin, Red Onion	0.22094	0.8311	126
Pumpkin, Red Cabbage	2.479	0.0142	126
Pumpkin. Spinach	58.313	0.006	126
Apple, Red Onion	21.108	0.0076	126
Apple, Red Cabbage	14.63	0.0086	126
Apple, Spinach	36.096	0.0072	126
Red Onion, Red Cabbage	2.3681	0.023	126
Red Onion, Spinach	30.491	0.0079	126
Red Cabbage, Spinach	19.941	0.008	126

Within level '30' of factor 'Temp'
Within level 'N' of factor 'RH'

Groups	t	P(perm)	Unique perms
CONTROL, Pumpkin	676.42	0.008	138
CONTROL, Apple	1108.2	0.0005	149
CONTROL, Red Onion	557.85	0.0068	129
CONTROL, Red Cabbage	686.16	0.0067	137
CONTROL, Spinach	2334.4	0.002	148
Pumpkin, Apple	1187.5	0.0019	151
Pumpkin, Red Onion	168.95	0.0093	126
Pumpkin, Red Cabbage	100.55	0.0083	126
Pumpkin. Spinach	1663.2	0.0013	138
Apple, Red Onion	821.51	0.001	109
Apple, Red Cabbage	1101.7	0.0054	144
Apple, Spinach	1667.1	0.0038	161
Red Onion, Red Cabbage	90.331	0.0086	126
Red Onion, Spinach	1066.1	0.0045	149
Red Cabbage, Spinach	1486.5	0.0012	161

PAIR-WISE TESTS **Term 'SuxTexRH' for pairs of levels of factor 'Temp'**

Within level 'CONTROL' of factor 'Subs'
Within level '70' of factor 'RH'

Within level 'CONTROL' of factor 'Subs'
Within level 'N' of factor 'RH'

Groups	t	P(perm)	Unique perms
20, 25	8.4758	0.0077	126
20, 30	6.8031	0.0092	126
25, 30	3.4756	0.0265	126

Within level 'Pumpkin' of factor 'Subs'
Within level '70' of factor 'RH'

Groups	t	P(perm)	Unique perms
20, 25	1.2919	0.2649	126
20, 30	1.8496	0.1027	126
25, 30	1.0786	0.3004	126

Within level 'Apple' of factor 'Subs'
Within level '70' of factor 'RH'

Groups	t	P(perm)	Unique perms
20, 25	5.1027	0.0091	126
20, 30	2.1937	0.096	126
25, 30	5.7405	0.007	126

Within level 'Red Onion' of factor 'Subs'
Within level '70' of factor 'RH'

Groups	t	P(perm)	Unique perms
20, 25	0.14509	0.8555	126
20, 30	3.1556	0.024	126
25, 30	3.63	0.0162	126

Within level 'Red Cabbage' of factor 'Subs'
Within level '70' of factor 'RH'

Groups	t	P(perm)	Unique perms
20, 25	3.5562	0.0147	126
20, 30	1.4534	0.1908	126
25, 30	1.5086	0.1479	126

Within level 'Spinach' of factor 'Subs'
Within level '70' of factor 'RH'

Groups	t	P(perm)	Unique perms
20, 25	0.89054	0.4611	126
20, 30	11.754	0.0079	126
25, 30	8.3289	0.01	126

Groups	t	P(perm)	Unique perms
20, 25	21.886	0.0093	126
20, 30	36.779	0.0078	126
25, 30	8.1696	0.0073	126

Within level 'Pumpkin' of factor 'Subs'
Within level 'N' of factor 'RH'

Groups	t	P(perm)	Unique perms
20, 25	273.72	0.0068	126
20, 30	36.627	0.0086	126
25, 30	162.59	0.0088	126

Within level 'Apple' of factor 'Subs'
Within level 'N' of factor 'RH'

Groups	t	P(perm)	Unique perms
20, 25	80.046	0.0081	126
20, 30	217.42	0.0107	126
25, 30	32.476	0.0076	126

Within level 'Red Onion' of factor 'Subs'
Within level 'N' of factor 'RH'

Groups	t	P(perm)	Unique perms
20, 25	1.1949	0.3157	126
20, 30	2.483	0.048	126
25, 30	64.528	0.0086	126

Within level 'Red Cabbage' of factor 'Subs'
Within level 'N' of factor 'RH'

Groups	t	P(perm)	Unique perms
20, 25	5.8283	0.0069	126
20, 30	13.95	0.0059	126
25, 30	23.551	0.0079	126

Within level 'Spinach' of factor 'Subs'
Within level 'N' of factor 'RH'

Groups	t	P(perm)	Unique perms
20, 25	430.75	0.0072	128
20, 30	148.01	0.0088	126
25, 30	846.34	0.0078	142

PAIR-WISE TESTS **Term 'SuxTexRH' for pairs of levels of factor 'RH'**

Within level 'CONTROL' of factor 'Subs'
Within level '20' of factor 'Temp'

Groups	t	P(perm)	Unique perms
70. N	8.8879	0.0075	126

Within level 'CONTROL' of factor 'Subs'
Within level '30' of factor 'Temp'

Groups	t	P(perm)	Unique perms
70. N	21.496	0.0084	126

Within level 'Pumpkin' of factor 'Subs'
Within level '25' of factor 'Temp'

Groups	t	P(perm)	Unique perms
70. N	21.695	0.0095	126

Within level 'Apple' of factor 'Subs'
Within level '20' of factor 'Temp'

Groups	t	P(perm)	Unique perms
70. N	0.13769	0.8391	126

Within level 'CONTROL' of factor 'Subs'
Within level '25' of factor 'Temp'

Groups	t	P(perm)	Unique perms
70. N	10.342	0.0082	126

Within level 'Pumpkin' of factor 'Subs'
Within level '20' of factor 'Temp'

Groups	t	P(perm)	Unique perms
70. N	11.956	0.0078	126

Within level 'Pumpkin' of factor 'Subs'
Within level '30' of factor 'Temp'

Groups	t	P(perm)	Unique perms
70. N	22.82	0.0084	126

Within level 'Apple' of factor 'Subs'
Within level '25' of factor 'Temp'

Groups	t	P(perm)	Unique perms
70. N	24.128	0.0079	126

Within level 'Apple' of factor 'Subs'
Within level '30' of factor 'Temp'

Groups	t	P(perm)	Unique perms
70. N	16.529	0.0081	126

Within level 'Red Onion' of factor 'Subs'
Within level '20' of factor 'Temp'

Groups	t	P(perm)	Unique perms
70. N	8.684	0.0069	126

Within level 'Red Onion' of factor 'Subs'
Within level '25' of factor 'Temp'

Groups	t	P(perm)	Unique perms
70. N	12.553	0.0081	126

Within level 'Red Onion' of factor 'Subs'
Within level '30' of factor 'Temp'

Groups	t	P(perm)	Unique perms
70. N	7.2207	0.0072	126

Within level 'Red Cabbage' of factor 'Subs'
Within level '20' of factor 'Temp'

Groups	t	P(perm)	Unique perms
70. N	6.1963	0.0062	126

Within level 'Red Cabbage' of factor 'Subs'
Within level '25' of factor 'Temp'

Groups	t	P(perm)	Unique perms
70. N	22.401	0.0088	126

Within level 'Red Cabbage' of factor 'Subs'
Within level '30' of factor 'Temp'

Groups	t	P(perm)	Unique perms
70. N	8.0031	0.0091	126

Within level 'Spinach' of factor 'Subs'
Within level '20' of factor 'Temp'

Groups	t	P(perm)	Unique perms
70. N	0.68739	0.7307	126

Within level 'Spinach' of factor 'Subs'
Within level '25' of factor 'Temp'

Groups	t	P(perm)	Unique perms
70. N	20.721	0.0105	126

Within level 'Spinach' of factor 'Subs'
Within level '30' of factor 'Temp'

Groups	t	P(perm)	Unique perms
70. N	7.5955	0.0074	126

PERMANOVA — Efiiciency of Conversion of Digested Feed (ECD)

Factors

**Name**	**Abbrev.**	**Type**	**Levels**
Subs	Su	Fixed	6
Temp	Te	Fixed	3
RH	RH	Fixed	2

PERMANOVA table of results

Source	df	SS	MS	Pseudo-F	P(perm)	Unique perms
Su	5	0.68002	0.136	944.85	0.0001	9942
Te	2	0.12592	0.062962	437.41	0.0001	9953
RH	1	0.059405	0.059405	412.7	0.0001	9825
SuxTe	10	0.041993	0.0041993	29.173	0.0001	9920
SuxRH	5	0.046755	0.0093511	64.964	0.0001	9953
TexRH	2	0.00074347	0.00037174	2.5825	0.0761	9955
SuxTexRH	10	0.028826	0.0028826	20.026	0.0001	9924
Res	144	0.020728	0.00014394			
Total	179	1.0044				

PAIR-WISE TESTS — **Term 'SuxTexRH' for pairs of levels of factor 'Subs'**

Within level '20' of factor 'Temp'
Within level '70' of factor 'RH'

Groups	t	P(perm)	Unique perms
CONTROL, Pumpkin	13.133	0.0079	126
CONTROL, Apple	7.3091	0.007	126
CONTROL, Red Onion	11.695	0.0084	126
CONTROL, Red Cabbage	8.5517	0.0095	126
CONTROL, Spinach	12.577	0.0093	126
Pumpkin, Apple	22.808	0.0082	126
Pumpkin, Red Onion	0.63316	0.5654	126
Pumpkin, Red Cabbage	3.5173	0.0248	126
Pumpkin. Spinach	27.668	0.0091	126
Apple, Red Onion	19.979	0.0099	126
Apple, Red Cabbage	16.082	0.0088	126
Apple, Spinach	14.842	0.0082	126

Within level '20' of factor 'Temp'
Within level 'N' of factor 'RH'

Groups	t	P(perm)	Unique perms
CONTROL, Pumpkin	3.5554	0.0071	126
CONTROL, Apple	2.7213	0.0464	126
CONTROL, Red Onion	0.24475	0.8501	126
CONTROL, Red Cabbage	0.69122	0.5149	126
CONTROL, Spinach	3.8633	0.0077	126
Pumpkin, Apple	26.522	0.008	126
Pumpkin, Red Onion	15.772	0.0074	126
Pumpkin, Red Cabbage	18.117	0.0086	126
Pumpkin. Spinach	31.476	0.0079	126
Apple, Red Onion	27.101	0.0079	126
Apple, Red Cabbage	29.369	0.0072	126
Apple, Spinach	17.303	0.0087	126

Red Onion, Red Cabbage	2.7562	0.031	126
Red Onion, Spinach	24.265	0.0086	126
Red Cabbage, Spinach	20.245	0.0084	126

Red Onion, Red Cabbage	4.9759	0.0072	126
Red Onion, Spinach	41.49	0.0083	126
Red Cabbage, Spinach	49.948	0.0093	126

Within level '25' of factor 'Temp'
Within level '70' of factor 'RH'

Groups	t	P(perm)	Unique perms
CONTROL, Pumpkin	13.944	0.0082	126
CONTROL, Apple	26.881	0.0058	126
CONTROL, Red Onion	5.4154	0.0086	126
CONTROL, Red Cabbage	5.0777	0.0087	126
CONTROL, Spinach	36.95	0.0089	126
Pumpkin, Apple	66.746	0.0073	126
Pumpkin, Red Onion	0.98116	0.41	126
Pumpkin, Red Cabbage	5.0326	0.0074	126
Pumpkin. Spinach	81.498	0.0091	126
Apple, Red Onion	17.446	0.0071	126
Apple, Red Cabbage	25.748	0.0077	126
Apple, Spinach	28.937	0.0079	126
Red Onion, Red Cabbage	1.9068	0.0819	126
Red Onion, Spinach	21.873	0.0074	126
Red Cabbage, Spinach	33.15	0.0091	126

Within level '25' of factor 'Temp'
Within level 'N' of factor 'RH'

Groups	t	P(perm)	Unique perms
CONTROL, Pumpkin	14.295	0.0073	126
CONTROL, Apple	4.6951	0.0076	126
CONTROL, Red Onion	10.777	0.0073	126
CONTROL, Red Cabbage	1.4374	0.188	126
CONTROL, Spinach	5.9055	0.0087	126
Pumpkin, Apple	34.886	0.0077	126
Pumpkin, Red Onion	6.5568	0.0074	126
Pumpkin, Red Cabbage	28.028	0.0082	126
Pumpkin. Spinach	38.905	0.0068	126
Apple, Red Onion	31.138	0.0075	126
Apple, Red Cabbage	18.133	0.0086	126
Apple, Spinach	2.7711	0.0327	126
Red Onion, Red Cabbage	23.224	0.0068	126
Red Onion, Spinach	35.746	0.0073	126
Red Cabbage, Spinach	25.652	0.0069	126

Within level '30' of factor 'Temp'
Within level '70' of factor 'RH'

Groups	t	P(perm)	Unique perms
CONTROL, Pumpkin	16.37	0.0074	126
CONTROL, Apple	18.727	0.0083	126
CONTROL, Red Onion	7.5445	0.0073	126
CONTROL, Red Cabbage	8.0807	0.008	126
CONTROL, Spinach	32.062	0.0095	126
Pumpkin, Apple	38.414	0.0087	126
Pumpkin, Red Onion	5.2026	0.0079	126
Pumpkin, Red Cabbage	2.0721	0.0876	126
Pumpkin. Spinach	50.294	0.0074	126
Apple, Red Onion	20.551	0.0082	126
Apple, Red Cabbage	17.543	0.0084	126
Apple, Spinach	42.34	0.0084	125
Red Onion, Red Cabbage	1.8828	0.094	126
Red Onion, Spinach	28.996	0.0089	126
Red Cabbage, Spinach	23.824	0.0085	126

Within level '30' of factor 'Temp'
Within level 'N' of factor 'RH'

Groups	t	P(perm)	Unique perms
CONTROL, Pumpkin	8.3482	0.0088	126
CONTROL, Apple	7.1282	0.0077	126
CONTROL, Red Onion	13.676	0.0081	126
CONTROL, Red Cabbage	8.5971	0.0097	126
CONTROL, Spinach	14.388	0.0075	126
Pumpkin, Apple	21.064	0.0084	126
Pumpkin, Red Onion	5.5745	0.0083	126
Pumpkin, Red Cabbage	2.1799	0.0916	126
Pumpkin. Spinach	28.983	0.0069	126
Apple, Red Onion	30.889	0.0089	126
Apple, Red Cabbage	46.107	0.007	126
Apple, Spinach	21.467	0.0087	126
Red Onion, Red Cabbage	10.058	0.0092	126
Red Onion, Spinach	38.931	0.0078	126
Red Cabbage, Spinach	56.166	0.0094	126

PAIR-WISE TESTS **Term 'SuxTexRH' for pairs of levels of factor 'Temp'**

Within level 'CONTROL' of factor 'Subs'
Within level '70' of factor 'RH'

Groups	t	P(perm)	Unique perms
20, 25	13.515	0.0075	126
20, 30	2.5461	0.0564	126
25, 30	15.16	0.0091	126

Within level 'CONTROL' of factor 'Subs'
Within level 'N' of factor 'RH'

Groups	t	P(perm)	Unique perms
20, 25	0.45894	0.6693	126
20, 30	1.8061	0.1189	126
25, 30	4.8088	0.0109	126

Within level 'Pumpkin' of factor 'Subs'
Within level '70' of factor 'RH'

Groups	t	P(perm)	Unique perms
20, 25	7.0287	0.0069	126
20, 30	3.2947	0.0157	126
25, 30	17.643	0.0076	126

Within level 'Pumpkin' of factor 'Subs'
Within level 'N' of factor 'RH'

Groups	t	P(perm)	Unique perms
20, 25	12.714	0.0077	126
20, 30	13.812	0.0079	126
25, 30	29.713	0.0077	126

Within level 'Apple' of factor 'Subs'
Within level '70' of factor 'RH'

Groups	t	P(perm)	Unique perms
20, 25	9.8593	0.0067	126
20, 30	0.92333	0.3862	126
25, 30	15.174	0.0073	126

Within level 'Apple' of factor 'Subs'
Within level 'N' of factor 'RH'

Groups	t	P(perm)	Unique perms
20, 25	6.0875	0.0076	126
20, 30	4.4581	0.0088	126
25, 30	8.0102	0.0085	126

Within level 'Red Onion' of factor 'Subs'

Within level 'Red Onion' of factor 'Subs'

Within level '70' of factor 'RH'

Groups	t	P(perm)	Unique perms
20, 25	3.7115	0.0076	126
20, 30	5.56	0.0072	126
25, 30	8.5083	0.0086	126

Within level 'Red Cabbage' of factor 'Subs'
Within level '70' of factor 'RH'

Groups	t	P(perm)	Unique perms
20, 25	5.5704	0.0068	126
20, 30	0.48907	0.6973	126
25, 30	6.5157	0.0086	126

Within level 'Spinach' of factor 'Subs'
Within level '70' of factor 'RH'

Groups	t	P(perm)	Unique perms
20, 25	2.2722	0.0677	126
20, 30	12.101	0.0087	126
25, 30	10.84	0.0081	126

PAIR-WISE TESTS **Term 'SuxTexRH' for pairs of levels of factor 'RH'**

Within level 'CONTROL' of factor 'Subs'
Within level '20' of factor 'Temp'

Groups	t	P(perm)	Unique perms
70. N	0.34683	0.7449	126

Within level 'CONTROL' of factor 'Subs'
Within level '30' of factor 'Temp'

Groups	t	P(perm)	Unique perms
70. N	10.401	0.0066	126

Within level 'Pumpkin' of factor 'Subs'
Within level '25' of factor 'Temp'

Groups	t	P(perm)	Unique perms
70. N	0.081213	0.9352	126

Within level 'Apple' of factor 'Subs'
Within level '20' of factor 'Temp'

Groups	t	P(perm)	Unique perms
70. N	2.1673	0.0676	126

Within level 'Apple' of factor 'Subs'
Within level '30' of factor 'Temp'

Groups	t	P(perm)	Unique perms
70. N	12.973	0.0072	126

Within level 'Red Onion' of factor 'Subs'
Within level '25' of factor 'Temp'

Groups	t	P(perm)	Unique perms
70. N	2.747	0.0431	126

Within level 'Red Cabbage' of factor 'Subs'
Within level '20' of factor 'Temp'

Groups	t	P(perm)	Unique perms
70. N	11.534	0.0079	126

Within level 'Red Cabbage' of factor 'Subs'
Within level '30' of factor 'Temp'

Within level 'N' of factor 'RH'

Groups	t	P(perm)	Unique perms
20, 25	29.401	0.0087	126
20, 30	8.1074	0.0068	126
25, 30	21.541	0.0078	126

Within level 'Red Cabbage' of factor 'Subs'
Within level 'N' of factor 'RH'

Groups	t	P(perm)	Unique perms
20, 25	24.558	0.0087	126
20, 30	4.4093	0.0166	126
25, 30	20.691	0.0076	126

Within level 'Spinach' of factor 'Subs'
Within level 'N' of factor 'RH'

Groups	t	P(perm)	Unique perms
20, 25	12.225	0.0079	126
20, 30	5.6404	0.0089	126
25, 30	14.799	0.0079	126

Within level 'CONTROL' of factor 'Subs'
Within level '25' of factor 'Temp'

Groups	t	P(perm)	Unique perms
70. N	7.6841	0.0088	126

Within level 'Pumpkin' of factor 'Subs'
Within level '20' of factor 'Temp'

Groups	t	P(perm)	Unique perms
70. N	4.1626	0.008	126

Within level 'Pumpkin' of factor 'Subs'
Within level '30' of factor 'Temp'

Groups	t	P(perm)	Unique perms
70. N	19.621	0.0082	126

Within level 'Apple' of factor 'Subs'
Within level '25' of factor 'Temp'

Groups	t	P(perm)	Unique perms
70. N	1.6814	0.1414	126

Within level 'Red Onion' of factor 'Subs'
Within level '20' of factor 'Temp'

Groups	t	P(perm)	Unique perms
70. N	14.092	0.0082	126

Within level 'Red Onion' of factor 'Subs'
Within level '30' of factor 'Temp'

Groups	t	P(perm)	Unique perms
70. N	6.1349	0.0067	126

Within level 'Red Cabbage' of factor 'Subs'
Within level '25' of factor 'Temp'

Groups	t	P(perm)	Unique perms
70. N	16.805	0.008	126

Within level 'Spinach' of factor 'Subs'
Within level '20' of factor 'Temp'

Groups	t	P(perm)	Unique perms
70. N	11.344	0.0073	126

Groups	t	P(perm)	Unique perms
70. N	0.63881	0.5062	126

Within level 'Spinach' of factor 'Subs'
Within level '25' of factor 'Temp'

Groups	t	P(perm)	Unique perms
70. N	10.813	0.0078	126

Within level 'Spinach' of factor 'Subs'
Within level '30' of factor 'Temp'

Groups	t	P(perm)	Unique perms
70. N	4.284	0.0069	126

PERMANOVA Growth Rate (GR)

Factors

**Name**	**Abbrev.**	**Type**	**Levels**
Subs	Su	Fixed	6
Temp	Te	Fixed	3
RH	RH	Fixed	2

PERMANOVA table of results

Source	df	SS	MS	Pseudo-F	P(perm)	Unique perms
Su	5	6.15E-05	1.23E-05	748.63	0.0001	9941
Te	2	5.67E-06	2.83E-06	172.57	0.0001	9956
RH	1	4.33E-06	4.33E-06	263.86	0.0001	9837
SuxTe	10	3.43E-06	3.43E-07	20.872	0.0001	9942
SuxRH	5	2.67E-06	5.34E-07	32.487	0.0001	9952
TexRH	2	3.01E-07	1.51E-07	9.1748	0.0002	9937
SuxTexRH	10	2.55E-06	2.55E-07	15.526	0.0001	9950
Res	144	2.37E-06	1.64E-08			
Total	179	8.28E-05				

PAIR-WISE TESTS **Term 'SuxTexRH' for pairs of levels of factor 'Subs'**

Within level '20' of factor 'Temp'
Within level '70' of factor 'RH'

Groups	t	P(perm)	Unique perms
CONTROL, Pumpkin	2.2523	0.0709	126
CONTROL, Apple	14.411	0.0069	126
CONTROL, Red Onion	2.1566	0.0625	126
CONTROL, Red Cabbage	3.3652	0.0136	126
CONTROL, Spinach	7.3799	0.0082	126
Pumpkin, Apple	15.093	0.0073	126
Pumpkin, Red Onion	4.4172	0.0075	126
Pumpkin, Red Cabbage	5.6074	0.0091	126
Pumpkin. Spinach	9.0357	0.0088	126
Apple, Red Onion	15.788	0.0079	126
Apple, Red Cabbage	18.954	0.0084	126
Apple, Spinach	6.0108	0.0077	126
Red Onion, Red Cabbage	1.2566	0.265	126
Red Onion, Spinach	6.2175	0.007	126
Red Cabbage, Spinach	5.7865	0.0075	126

Within level '20' of factor 'Temp'
Within level 'N' of factor 'RH'

Groups	t	P(perm)	Unique perms
CONTROL, Pumpkin	2.2664	0.0759	126
CONTROL, Apple	10.185	0.0075	126
CONTROL, Red Onion	1.6495	0.1532	126
CONTROL, Red Cabbage	2.5688	0.0315	126
CONTROL, Spinach	10.206	0.0076	126
Pumpkin, Apple	23.551	0.0078	126
Pumpkin, Red Onion	6.0616	0.0079	126
Pumpkin, Red Cabbage	9.0197	0.0088	126
Pumpkin. Spinach	23.49	0.0077	126
Apple, Red Onion	17.041	0.0067	126
Apple, Red Cabbage	36.456	0.0074	126
Apple, Spinach	0.39333	0.6765	126
Red Onion, Red Cabbage	1.4872	0.1646	126
Red Onion, Spinach	17.002	0.0092	126
Red Cabbage, Spinach	35.326	0.0082	126

Within level '25' of factor 'Temp'
Within level '70' of factor 'RH'

Groups	t	P(perm)	Unique perms
CONTROL, Pumpkin	26.79	0.0083	126
CONTROL, Apple	30.869	0.0076	126
CONTROL, Red Onion	10.915	0.0096	126
CONTROL, Red Cabbage	9.5166	0.0083	126
CONTROL, Spinach	27.911	0.0074	126
Pumpkin, Apple	72.448	0.0079	126
Pumpkin, Red Onion	11.82	0.0082	126
Pumpkin, Red Cabbage	5.7087	0.0088	126
Pumpkin. Spinach	60.926	0.0079	125
Apple, Red Onion	39.212	0.0091	126
Apple, Red Cabbage	26.421	0.0075	126
Apple, Spinach	1.9476	0.0907	126
Red Onion, Red Cabbage	1.9381	0.0972	126
Red Onion, Spinach	36.235	0.0091	126
Red Cabbage, Spinach	26.048	0.0079	126

Within level '25' of factor 'Temp'
Within level 'N' of factor 'RH'

Groups	t	P(perm)	Unique perms
CONTROL, Pumpkin	6.2225	0.0078	126
CONTROL, Apple	27.326	0.0089	126
CONTROL, Red Onion	0.31099	0.6477	126
CONTROL, Red Cabbage	5.434	0.0076	126
CONTROL, Spinach	5.5099	0.0084	126
Pumpkin, Apple	48.179	0.0088	126
Pumpkin, Red Onion	6.711	0.007	126
Pumpkin, Red Cabbage	1.0942	0.2662	126
Pumpkin. Spinach	8.3758	0.0084	126
Apple, Red Onion	27.372	0.0097	126
Apple, Red Cabbage	46.534	0.0075	126
Apple, Spinach	5.8873	0.009	126
Red Onion, Red Cabbage	5.9062	0.009	126
Red Onion, Spinach	5.3706	0.0083	126
Red Cabbage, Spinach	8.0503	0.0072	126

Within level '30' of factor 'Temp'
Within level '70' of factor 'RH'

Groups	t	P(perm)	Unique perms
CONTROL, Pumpkin	12.74	0.0088	126
CONTROL, Apple	28.863	0.0088	126
CONTROL, Red Onion	6.3811	0.0072	126
CONTROL, Red Cabbage	1.9911	0.0626	126
CONTROL, Spinach	17.595	0.0084	126
Pumpkin, Apple	32.368	0.0088	125
Pumpkin, Red Onion	3.8997	0.0169	126
Pumpkin, Red Cabbage	7.7739	0.0069	125
Pumpkin. Spinach	25.389	0.0071	126
Apple, Red Onion	21.574	0.0077	126
Apple, Red Cabbage	16.945	0.0095	126
Apple, Spinach	1.0968	0.291	126
Red Onion, Red Cabbage	3.5014	0.0324	126
Red Onion, Spinach	17.873	0.0075	126
Red Cabbage, Spinach	13.848	0.0079	126

Within level '30' of factor 'Temp'
Within level 'N' of factor 'RH'

Groups	t	P(perm)	Unique perms
CONTROL, Pumpkin	2.8718	0.0265	126
CONTROL, Apple	14.644	0.0094	126
CONTROL, Red Onion	3.3547	0.0077	126
CONTROL, Red Cabbage	7.1656	0.0099	126
CONTROL, Spinach	9.9115	0.0081	126
Pumpkin, Apple	27.64	0.0084	126
Pumpkin, Red Onion	2.2457	0.0336	126
Pumpkin, Red Cabbage	5.7427	0.0078	126
Pumpkin. Spinach	15.371	0.0079	126
Apple, Red Onion	9.0584	0.0087	126
Apple, Red Cabbage	77.854	0.0055	126
Apple, Spinach	2.003	0.08	126
Red Onion, Red Cabbage	0.8295	0.5909	126
Red Onion, Spinach	8.0296	0.0082	126
Red Cabbage, Spinach	22.922	0.0092	126

PAIR-WISE TESTS **Term 'SuxTexRH' for pairs of levels of factor 'Temp'**

Within level 'CONTROL' of factor 'Subs'
Within level '70' of factor 'RH'

Groups	t	P(perm)	Unique perms
20, 25	0.82692	0.5449	126
20, 30	1.5009	0.168	126
25, 30	1.3573	0.2043	126

Within level 'CONTROL' of factor 'Subs'
Within level 'N' of factor 'RH'

Groups	t	P(perm)	Unique perms
20, 25	1.2458	0.271	126
20, 30	0.51627	0.6082	125
25, 30	2.8982	0.0078	126

Within level 'Pumpkin' of factor 'Subs'
Within level '70' of factor 'RH'

Groups	t	P(perm)	Unique perms
20, 25	7.6666	0.0086	126
20, 30	6.6209	0.0094	126
25, 30	0.072066	0.9506	126

Within level 'Pumpkin' of factor 'Subs'
Within level 'N' of factor 'RH'

Groups	t	P(perm)	Unique perms
20, 25	1.8681	0.0717	126
20, 30	1.8736	0.092	125
25, 30	5.5909	0.0091	126

Within level 'Apple' of factor 'Subs'
Within level '70' of factor 'RH'

Groups	t	P(perm)	Unique perms
20, 25	16.202	0.0086	126
20, 30	14.888	0.0097	126
25, 30	3.8534	0.0164	125

Within level 'Apple' of factor 'Subs'
Within level 'N' of factor 'RH'

Groups	t	P(perm)	Unique perms
20, 25	5.5299	0.0084	126
20, 30	12.777	0.0076	126
25, 30	0.37861	0.7055	126

Within level 'Red Onion' of factor 'Subs'
Within level '70' of factor 'RH'

Groups	t	P(perm)	Unique perms
20, 25	11.185	0.0062	126
20, 30	9.2089	0.0071	126
25, 30	1.3176	0.2082	126

Within level 'Red Onion' of factor 'Subs'
Within level 'N' of factor 'RH'

Groups	t	P(perm)	Unique perms
20, 25	5.1424	0.0062	126
20, 30	4.1818	0.0087	126
25, 30	2.3777	0.0222	126

Within level 'Red Cabbage' of factor 'Subs'
Within level '70' of factor 'RH'

Groups	t	P(perm)	Unique perms
20, 25	13.619	0.0086	126
20, 30	7.0857	0.0088	126
25, 30	4.1534	0.0147	126

Within level 'Red Cabbage' of factor 'Subs'
Within level 'N' of factor 'RH'

Groups	t	P(perm)	Unique perms
20, 25	21.889	0.0083	126
20, 30	25.269	0.0079	126
25, 30	1.1086	0.2848	126

Within level 'Spinach' of factor 'Subs'
Within level '70' of factor 'RH'

Groups	t	P(perm)	Unique perms
20, 25	2.4307	0.0391	126
20, 30	1.6222	0.1337	126
25, 30	0.76452	0.4543	126

Within level 'Spinach' of factor 'Subs'
Within level 'N' of factor 'RH'

Groups	t	P(perm)	Unique perms
20, 25	7.3139	0.007	126
20, 30	4.4846	0.0066	126
25, 30	4.1987	0.0098	126

PAIR-WISE TESTS	Term 'SuxTexRH' for pairs of levels of factor 'RH'		

Within level 'CONTROL' of factor 'Subs'
Within level '20' of factor 'Temp'

Groups	t	P(perm)	Unique perms
70. N	1.5023	0.1673	126

Within level 'CONTROL' of factor 'Subs'
Within level '25' of factor 'Temp'

Groups	t	P(perm)	Unique perms
70. N	3.1513	0.0225	126

Within level 'CONTROL' of factor 'Subs'
Within level '30' of factor 'Temp'

Groups	t	P(perm)	Unique perms
70. N	5.7519	0.0066	126

Within level 'Pumpkin' of factor 'Subs'
Within level '20' of factor 'Temp'

Groups	t	P(perm)	Unique perms
70. N	2.0398	0.0832	126

Within level 'Pumpkin' of factor 'Subs'
Within level '25' of factor 'Temp'

Groups	t	P(perm)	Unique perms
70. N	30.488	0.0066	126

Within level 'Pumpkin' of factor 'Subs'
Within level '30' of factor 'Temp'

Groups	t	P(perm)	Unique perms
70. N	15.548	0.009	126

Within level 'Apple' of factor 'Subs'
Within level '20' of factor 'Temp'

Groups	t	P(perm)	Unique perms
70. N	0.0093586	1	126

Within level 'Apple' of factor 'Subs'
Within level '25' of factor 'Temp'

Groups	t	P(perm)	Unique perms
70. N	7.7133	0.0078	126

Within level 'Apple' of factor 'Subs'
Within level '30' of factor 'Temp'

Groups	t	P(perm)	Unique perms
70. N	8.6912	0.0088	126

Within level 'Red Onion' of factor 'Subs'
Within level '20' of factor 'Temp'

Groups	t	P(perm)	Unique perms
70. N	1.9726	0.0929	126

Within level 'Red Onion' of factor 'Subs'
Within level '25' of factor 'Temp'

Groups	t	P(perm)	Unique perms
70. N	13.626	0.0078	126

Within level 'Red Onion' of factor 'Subs'
Within level '30' of factor 'Temp'

Groups	t	P(perm)	Unique perms
70. N	2.2721	0.0597	126

Within level 'Red Cabbage' of factor 'Subs'
Within level '20' of factor 'Temp'

Groups	t	P(perm)	Unique perms
70. N	2.7424	0.0145	126

Within level 'Red Cabbage' of factor 'Subs'
Within level '25' of factor 'Temp'

Groups	t	P(perm)	Unique perms
70. N	9.0347	0.0078	126

Within level 'Red Cabbage' of factor 'Subs'
Within level '30' of factor 'Temp'

Groups	t	P(perm)	Unique perms
70. N	1.8188	0.0152	126

Within level 'Spinach' of factor 'Subs'
Within level '20' of factor 'Temp'

Groups	t	P(perm)	Unique perms
70. N	6.109	0.0083	126

Within level 'Spinach' of factor 'Subs'
Within level '25' of factor 'Temp'

Groups	t	P(perm)	Unique perms
70. N	4.374	0.0074	126

Within level 'Spinach' of factor 'Subs'
Within level '30' of factor 'Temp'

Groups	t	P(perm)	Unique perms
70. N	0.94827	0.3948	126
